# *Myceliophthora thermophila* M77 utilizes hydrolytic and oxidative mechanisms to deconstruct biomass

**DOI:** 10.1186/s13568-016-0276-y

**Published:** 2016-11-02

**Authors:** Hévila Brognaro dos Santos, Thaís Milena Souza Bezerra, José G. C. Pradella, Priscila Delabona, Deise Lima, Eleni Gomes, Steve D. Hartson, Janet Rogers, Brian Couger, Rolf Prade

**Affiliations:** 1 Instituto de Física de São Carlos, Universidade de São Paulo, São Carlos, SP Brazil; 2 Laboratório de Microbiologia e Bioquímica Aplicada, Departamento de Biologia, IBILCE/UNESP, Rua Cristovão Colombo, 2265 Bairro Jd. Nazareth, São José do Rio Preto, SP CEP 15054-000 Brazil; 3 Laboratório Nacional de Ciência e Tecnologia do Bioetanol, Rua Giuseppe Máximo Scolfaro, 10.000, Bairro Guará, Campinas, SP CEP 13083-970 Brazil; 4 Laboratório de Enzimologia, Instituto de Química, UNESP, Araraquara, São Paulo Brazil; 5 Department of Microbiology and Molecular Genetics, Oklahoma State University, Stillwater, OK 74078 USA; 6 Department of Biochemistry and Molecular Biology, Oklahoma State University, Stillwater, OK 74078 USA

**Keywords:** *Myceliophthora thermophila*, Biomass, Cellulose degradation, Secretome composition, Cellulose hydrolysis, Cellulose oxidation

## Abstract

Biomass is abundant, renewable and useful for biofuel production as well as chemical priming for plastics and composites. Deconstruction of biomass by enzymes is perceived as recalcitrant while an inclusive breakdown mechanism remains to be discovered. Fungi such as *Myceliophthora thermophila* M77 appear to decompose natural biomass sources quite well. This work reports on this fungus fermentation property while producing cellulolytic enzymes using natural biomass substrates. Little hydrolytic activity was detected, insufficient to explain the large amount of biomass depleted in the process. Furthermore, this work makes a comprehensive account of extracellular proteins and describes how secretomes redirect their qualitative protein content based on the nature and chemistry of the nutritional source. Fungus grown on purified cellulose or on natural biomass produced secretomes constituted by: cellobiohydrolases, cellobiose dehydrogenase, β-1,3 glucanase, β-glucosidases, aldose epimerase, glyoxal oxidase, GH74 xyloglucanase, galactosidase, aldolactonase and polysaccharide monooxygenases. Fungus grown on a mixture of purified hemicellulose fractions (xylans, arabinans and arabinoxylans) produced many enzymes, some of which are listed here: xylosidase, mixed β-1,3(4) glucanase, β-1,3 glucanases, β-glucosidases, β-mannosidase, β-glucosidases, galactosidase, chitinases, polysaccharide lyase, endo β-1,6 galactanase and aldose epimerase. Secretomes produced on natural biomass displayed a comprehensive set of enzymes involved in hydrolysis and oxidation of cellulose, hemicellulose-pectin and lignin. The participation of oxidation reactions coupled to lignin decomposition in the breakdown of natural biomass may explain the discrepancy observed for cellulose decomposition in relation to natural biomass fermentation experiments.

**Electronic supplementary material:**

The online version of this article (doi:10.1186/s13568-016-0276-y) contains supplementary material, which is available to authorized users.

## Introduction

Lignocellulosic biomass polymers are a massive and renewable source for production of biofuels and biochemicals, because they trap about 60% of all sugars produced by plants on earth. Just as it happens in nature, man-made lignocellulosic biomass such as corn stover and sugar cane bagasse that pile up alongside bio refineries and could be broken down enzymatically (Amorim et al. 2011; Lal 2005). However, currently the cost of cellulase enzyme cocktails are the bottleneck to the economical production of these second generation biofuels (Phillips et al. 2011) mainly because enzymatic conversion of lignocellulose into sugars is a slow and recalcitrant process and cellulose is an insoluble crystalline substance (Himmel and Bayer 2009) clustered within phenolic lignin (benzene ether linkages) hindering its ability to be enzymatically processed (Lacayo et al. 2013).

For cellulase aided breakdown of cellulose to take place, a single chain must be separated from the crystalline fiber and fitted into an enzyme binding site where catalytic Asp or Glu residues hydrolyze through a general acid/base mechanism the glycoside bond (Divne et al. 1994). The disconnection of the glucan chain from crystalline cellulose fibers has been proposed to be the bottleneck in enzymatic hydrolysis of cellulose (Himmel and Bayer 2009).

This recalcitrance towards the degradation of cellulose is abundantly illustrated in the repertoire of cellulose degrading enzymes produced by microorganisms that try to use this polymer as a carbon source (Segato et al. 2014). Most microorganisms produce at least three types of glycosidic bond breaking enzymes; cellobiohydrolases (also defined as exo glucanases and/or processive glucanases), endo glucanases and β-glucosidases. For comprehensive reviews of hydrolytic biomass breakdown refer to (Benz et al. 2013; Coutinho et al. 2009; Glass et al. 2013; Martens-Uzunova and Schaap 2009; Segato et al. 2014).

Recently oxidoreductase enzymes such as polysaccharide monooxygenases (PMO’s) that directly oxidize glycoside bonds generating aldones and lactones have been discovered highlighting the role of oxidation reactions in the breakdown of biomass components (Beeson et al. 2012; Horn et al. 2012; Langston et al. 2011; Quinlan et al. 2011; Vaaje-Kolstad et al. 2010).

A direct role of cellobiose dehydrogenase on cellulose depolymerization via the oxidation of glycoside bonds aided by Fenton chemistry has been suggested (Canevascini et al. 1991; Divne et al. 1994; Henriksson et al. 2000a; Mansfield et al. 1997; Mason et al. 2003; Stahlberg et al. 1996; Westermark and Eriksson 1975; Zamocky et al. 2006). Moreover, the participation of cellobiose dehydrogenase in oxidation of other biomass components such as lignin has also been considered (Henriksson et al. 2000b; Hilden et al. 2000).

Here we report on the efficiency of biomass bioconversion by *Myceliophthora thermophila* M77, whereas in a traditional bioreactor, the fungus completely consumes biomass sources (sugar cane bagasse) but shows little cellulase filter paper activity, leading the research to determine global secretome composition of *M. thermophila* growing on biomass and purified biomass components (cellulose and hemicellulose). When purified cellulose was available, the fungus produced a secretome that included hydrolytic and oxidative enzymes, almost exclusively dedicated to the breakdown of cellulose and cellulose related molecules. When natural biomass was available, the fungus produced a comprehensive collection of enzymes in addition to cellobiose dehydrogenase involved in oxidation and hydrolysis of cellulose, hemicellulose-pectin and lignin.

## Materials and methods

### Strains, media, solutions and biomass sources

The strain used in this work *M. thermophila* M77 was isolated from a sugar cane bagasse pile of the northwest region of São Paulo State, Brazil and was deposited at the Fungal Genetics Stock Center FGSC# 26436 (Moretti et al. 2012). A similar *M. thermophila* strain ATCC 42464 was recently sequenced by the DOE Joint Genome Institute Fungal Genomics Program (Berka et al. 2011; Kolbusz et al. 2014) and was used for DNA sequence based interpretation of LC–MS/MS data.

*Myceliophthora thermophila* M77 was grown on 1.8% agar petri dishes in Mandels and Sternberg salts (Mandels and Sternberg 1976) amended with 1.0% glucose and 0.1% peptone incubated at 45 °C for 7 days, or as otherwise stated. Spores were scraped off the plates with a platinum loop, suspended in 0.1% Tween 80 (Sigma-Aldrich, St Louis, MO, USA) and used to pre-inoculate (about 1 × 10^7^ spores/mL) shaker flasks incubated at 45 °C, 250 rpm for 72 h prior to direct transfer to a bioreactor vessel or a large-scale shaker flask experiment.

In experiments using biomass substrates and derivatives, the glucose was replaced with 1.0% (w/v) of commercial microcrystalline cellulose (EC) (Celuflok 200™, Celuflok Ind. Com. São Paulo, Brazil), “in natura” milled (200-μm particle size) sugar cane bagasse (SCBIN), lignin removed (sodium hydroxide extracted) and steam exploded sugar cane bagasse (SCBDL), steam exploded sugar cane bagasse only (SCBSE), wheat bran (WB), milled soybeans (SM), and fructooligosaccharides (FOS). Sugar cane bagasse sources were prepared and chemically defined as described in (Rocha et al. 2011) and milled powders washed with water and autoclaved prior to use.

### Shaken flask experiments

Twenty millilitre of pre-inoculum was added to 1 L Erlenmeyer flasks containing 200 mL of Mandels and Sternberg salts, 0.1% peptone amended with 1% (w/v) SCBSE, SCBDL, WB, EC, SM, glycerol (GLY), lactose (LAC), sucrose (SUC) and FOS alone or in combinations and proportions as indicated. Incubations were made in an orbital shaker (Innova 44R Stackable Incubator Shaker, New Brunswick, NJ, USA) for up to 120 h at 45 °C and 250 rpm and samples withdrawn daily for enzyme activity and protein quantifications.

### Bioreactor experiments

Bioreactor assays were performed in a lab-scale Bioflo^®^115 (New Brunswick, NJ, USA) with a working volume of 1.5 L, using SCBSE, WB and sucrose as carbon sources. The pre-inoculum was 10% of the final volume. Cultivations were conducted in batch or pulse-fed batch mode (as indicated in Figures and Tables), the dissolved O_2_ concentration was >30 % of air saturation and mechanical stirring was performed with two Rushton-type impellers, in the range of 200–400 rpm. Prior to use all equipment was sterilized for 30 min at 121 °C. Automatic pH control was done using a 0.4 M HCl and NH_4_OH aqueous solution 3:1 (v/v) and foaming was controlled as required by manual addition of sterile antifoam polypropylene glycol 2000 (Dow Chemical, São Paulo, Brazil). Samples were withdrawn under sterile conditions daily, centrifuged at 12,000 rpm for 25 min at 4 °C and supernatants collected for cellulase (FPase), xylanase, β-glucosidase activity and total protein quantification.

### Enzymatic activity assays

Cellulase activity was determined by the method of Ghose (1987) that measures the release of detectable reducing sugars removed from filter paper (FPase). Xylanase activity was determined by the method described by Bailey and Poutanen (1989). Both FPase and xylanase activities were performed measuring reducing sugars by the dinitrosalicylic acid (DNS) method (Miller 1959), using glucose and xylose standards as appropriate. β-glucosidase and cellobiohydrolase was measured using *p*-nitrophenol-β-d-glucopyranoside (pNPG) and *p*-nitrophenol-β-d-cellobioside (pNPC) (Sigma-Aldrich, USA) as substrate, respectively (Zhang et al. 2009). Total protein content was measured in micro plates using the Bio-Rad assay reagent (Bio-Rad Laboratories, Hercules, USA), using a procedure based on the Bradford method (Bradford 1976) with bovine serum albumin as standard. One enzyme unit (IU) corresponded to the amount of product (μmol) produced per minute and cellulase activity was expressed as filter paper units (FPU) calculated according to (Ghose 1987). Cellobiose dehydrogenase activity was assayed through 2,6-dichlorophenol-indophenol (DCPIP) reduction. The decrease in absorbance was measured continuously at 520 nm (ε = 6.8 × 10^3^ M^−1^ cm^−1^) in sodium acetate buffer (50 mM; pH 5) containing DCPIP 0.3 mM, sodium lactate 30 mM and NaF 4 mM. One enzyme unit (IU) corresponded to the amount of enzyme reducing 1 μmol of DCPIP per minute (Baminger et al. 1999). Laccase activity was measured continuously by the oxidation rate of ABTS^2+^ to ABTS^●+^ at 420 nm (ε = 3.6 × 10^4^ M^−1^ cm^−1^) in acetate buffer (50 mM; pH 3.5) containing ABTS (5 mM) in a final volume of 2 mL at 25 °C. One enzyme unit (IU) corresponded to the amount of enzyme that oxidized 1 µmol of ABTS per minute (Bourbonnais et al. 1995).

### Enzymatic biomass hydrolysis

Bioconversion assays were conducted in 50 mL (125 mL Erlenmeyer flasks) final volumes, buffered with citrate 50 mM pH 5, 5% (w/v) of substrate (EC, SCBIN, SCBDL or SCBSE) and a protein load of 0.05 mg/g of glucan, incubated at 50 °C at 200 rpm. All experiments were performed in duplicates. Samples were withdrawn at 0, 6, 12, 24 and 48 h and the glucose, gluconic acid, cellobiose, cellobionic acid, xylose, arabinose, acetic, formic and levulinic acid concentrations were quantified by HPLC Dionex Ultimate 300 system equipped with a refractive index detector (HPLC-RI) using an Aminex ^®^HPX-87H column and eluted with 5 mM H_2_SO_4_ at 0.6 mL/min. Sugars and acids in control samples containing only the respective substrate and citrate buffer 50 mM pH 5.0 were also measured. All samples were filtered using a Millex TM 0.22 μm filter prior to further analysis.

### Production of secretomes

*Myceliophthora thermophila* M77 was grown in Erlenmeyer flasks on Mandels & Sternberg salts, 0.1% peptone containing SCBIN (natural sugar cane bagasse, milled at 200 μm particle size) as well as modified sugar cane bagasse versions such as SCBDL (delignified with sodium hydroxide), SCBSE (steam exploded), purified celluloses containing 0.5% of avicel and 0.5% carboxymethylcellulose (Sigma Aldrich, St Louis MO), purified hemicelluloses containing 0.2% of each; birchwood-, beechwood-, oat spelt-xylan, arabinan and arabinoxylan (Megazyme International, Wicklow, Ireland) and glucose (control).

Secreted proteins were collected after a 36 h cultivation period at 45 °C, 200 rpm supernatants cleared by centrifugation (5000×*g*), concentrated by ultra-filtration (10,000 MWCO, PES membrane, Vivaspin, Littleton USA), rinsed twice with 5 mL of sodium acetate buffer 50 mM pH 5 and the proteins were separated by SDS-PAGE (Weber and Osborn 1969).

### Secretome peptide mapping by liquid chromatography-tandem mass spectrometry (LC–MS/MS)

For secretome peptide mapping experiments two independent cultures and two protein separations through SDS-PAGE were carried out. For secretome LC–MS/MS analysis 20–30 μg of total secretome proteins were loaded onto an SDS-PAGE gel and while in Fig. 3 we show a fully resolved SDS-PAGE gel for proteomics experiments, for proteomics the SDS-PAGE was run for only about one inch into the 12% separation gel, stained with Comassie blue and the entire protein banding profile excised, processed for LC–MS/MS according to (Shevchenko et al. 1996) with modifications. Isolated gel bands were reduced with Tris (2-carboxyethyl) phosphine, alkylated by 2-Iodoacetamide, digested for 6–16 h with 8 μg/mL trypsin using ammonium bicarbonate buffer and analyzed by LC–MS/MS using LTQ-Orbitrap XL hybrid mass spectrometer (Thermo Scientific, Waltham, MA, USA). For this analysis, an Eksigent LC pump was used to separate peptide populations on analytical C18 nano-columns, with the column effluent being sprayed directly into a New Objective Picoview ion source. Using a “Top Three” MS/MS method, the Orbitrap analyzer collected accurate (5 ppm) scans of intact peptides for one second, at the same time as the LTQ ion trap simultaneously performed MS/MS fragmentation analysis of each of the three most abundant peptides eluting in that 1 s chromatographic fraction (0.8 Da mass accuracy).

The LC–MS/MS raw files were used for database Mascot (version 2.2.04, Matrix Science, London UK) searches run on a NCBI *M. thermophila* ATCC_42464 specific subset. The DNA and amino acid sequence of *M. thermophila* M77 are 98.95 and 99.45% identical to *M. thermophila* ATCC_42464, respectively. Searches were validated using Scaffold (version 4.0.7, Proteome Software Inc. Portland, OR) with a protein threshold of 5% FDR and a peptide threshold of 99%. Further management of spectral data were performed on downloaded Excel files, total spectral counts (TSC) were normalized (against the total spectral count of each sample) and finally duplicates averaged (Additional file 1: Table S1). Thus, the quantitative value NTSC (normalized total spectrum counts) for a given protein component of a secretome reflects the amount of protein secreted as a direct response to the applied carbon source.

## Results

### Bioreactors and shakers producing biomass-degrading enzymes

**Fig. 1:**
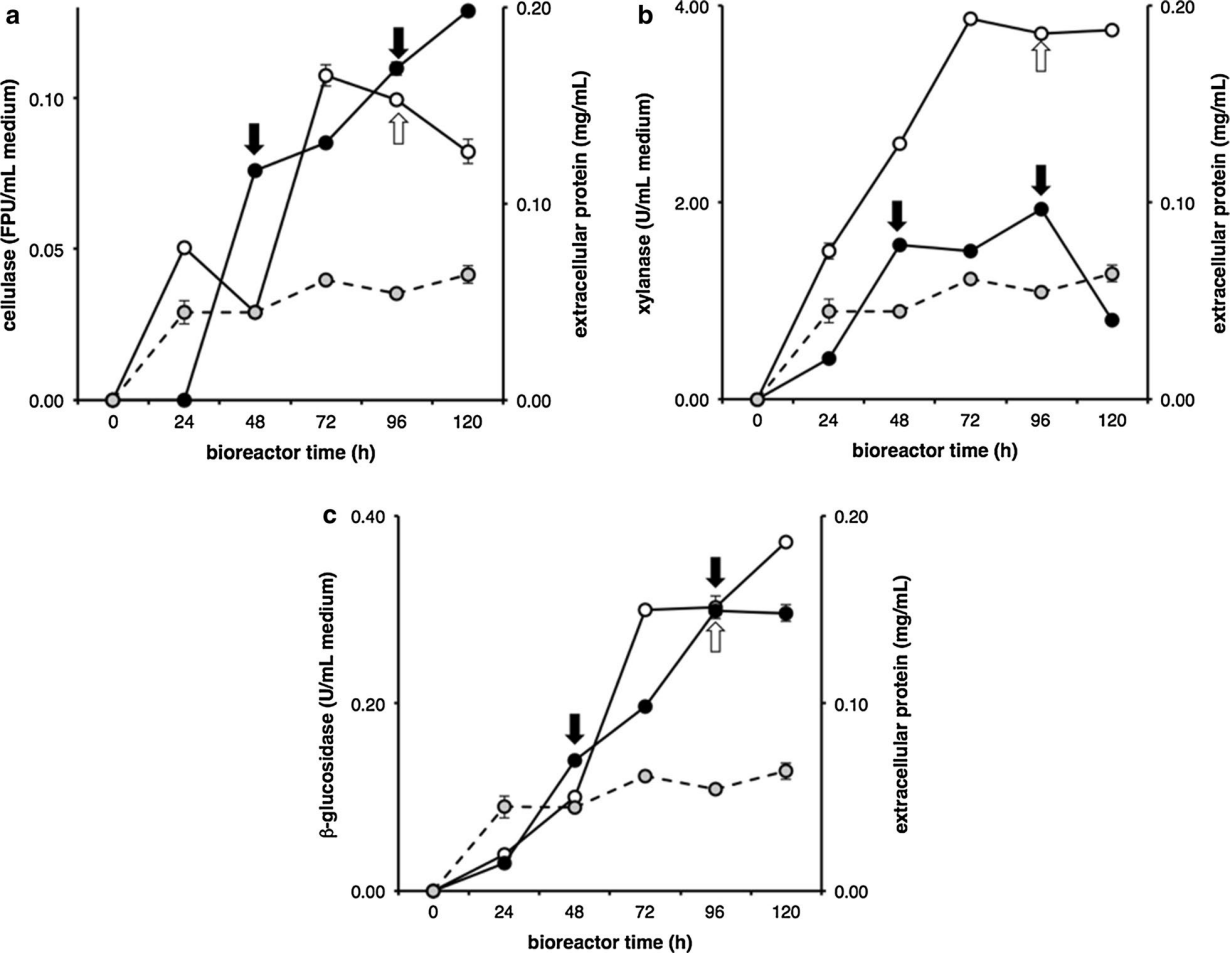
*Myceliophthora thermophila* M77 fed-batch bioreactor with steam exploded sugar cane bagasse (SCBSE) as the carbon source. Bioreactors containing Mandels and Sternberg salts amended with 0.1% peptone and 1% steam exploded sugar cane bagasse (SCBSE) were conducted for 120 h at 45 °C, constant pH 5.0 and feed-pulsed with SCBSE at the indicated time points (*arrows*). Extracellular protein accumulation (*shaded symbols*), cellulase (**a**), xylanase (**b**) and β-glucosidase (**c**) activities were followed in a single feed-pulse (*open symbols*) and a double feed-pulse (*closed symbols*) regimen

Figure 1 reports a typical bioreactor experiment in which the carbon source was SCBSE (steam-exploded sugar cane bagasse). Extracellular protein and expected enzyme activities, cellulase (measured as activity on filter paper (FPU), xylanase and β-glucosidase accumulated in the medium over time reaching a peak at or around 96 h.

Table 1 shows a series of shaking flask experiments modifying the forms of biomass and combinations with simple carbon sources such as glycerol, lactose, sucrose and glucose designed to improve enzyme production. The highest cellulase activity, as judged by the DNS assay, was observed with steam-exploded biomass (0.23 FPU/mL) while lignin extracted biomass showed lower cellulase activity (0.10 FPU/mL) and other sources such as wheat bran and purified cellulose as well as combinations thereof did not improve cellulase activity (Table 1). Thus, none of the biomass variants produced significant improvement over cellulase activity (FPU). For xylanase activity a similar picture occurs, none of the biomass derivatives improve drastically xylanase activity, however the addition of a non-repressive carbon source such as lactose (LAC), phospho-fructo-oligosacharides (FOS) or sucrose (SUC) resulted in a slight increase in xylanase activity (Table 1).

**Table 1:** *Myceliophthora thermophila* enzyme accumulation in shaking flask bioreactors

	Cellulase		Xylanase		β-glucosidase		Protein	
	FPU/mL	p (h)	IU/mL	p (h)	IU/mL	p (h)	mg/mL	p (h)
SCBSE	0.23 ± 0.03	48	2.90 ± 0.06	48	0.43 ± 0.10	72	0.12 ± 0.01	120
SCBDL	0.10 ± 0.01	48	2.60 ± 0.20	72	0.33 ± 0.06	96	0.07 ± 0.01	96
WB	0.12 ± 0.02	48	2.00 ± 0.04	24	1.00 ± 0.04	120	0.15 ± 0.02	120
EC	0.10 ± 0.01	48	2.60 ± 0.14	72	0.33 ± 0.08	96	0.04 ± 0.01	48
SCBSE + WB	0.10 ± 0.02	72	2.80 ± 0.05	72	0.55 ± 0.05	72	0.13 ± 0.02	96
SCBSE + SM	0.13 ± 0.02	72	2.50 ± 0.10	48	0.53 ± 0.07	120	0.24 ± 0.03	96
SCBSE + SM	0.13 ± 0.03	72	2.80 ± 0.08	72	0.42 ± 0.05	120	0.10 ± 0.01	120
3XSCBSE + 1XFOS	–		3.50 ± 0.05	48	–		0.11 ± 0.01	24
1XSCBSE + 1XLAC	–		3.50 ± 0.10	72	–		0.08 ± 0.01	24
3XSCBSE + 1XLAC	0.19 ± 0.02	72	3.10 ± 0.08	72	0.57 ± 0.05	120	0.12 ± 0.01	96
3XSCBSE + 1XGLY	0.18 ± 0.02	96	2.80 ± 0.05	72	0.46 ± 0.09	120	0.10 ± 0.02	48
3XSCBSE + 1XSUC	0.18 ± 0.04	96	3.30 ± 0.12	72	0.43 ± 0.08	120	0.10 ± 0.01	120

*SCBSE* steam-exploded sugar cane bagasse;* SCBDL * delignified steam-exploded sugar cane bagasse;* EC* celuflok 200™;* WB* wheat bran;* SM *soybean mill;* GLY* glycerol;* LAC* lactose;* SUC* sucrose;* FOS* commercial phosphofructooligosacharide. Bioreactor time (h) of peak activity (p)

Table 2 shows a series of six bioreactor runs in which we varied pH and temperature, feeding schedule as well as the combination of biomass sources, designed to overcome process side effects such as the possible interference of proteases and the onset of carbon catabolite repression. With the exception of the presence of sucrose (Table 2, run #5) that doubled the amount of cellulase, none of the other variations seemed to enhance filter paper activity.

**Table 2:** Substrate influence on enzyme accumulation in bioreactors

Run	Bioreactor conditions				Cellulase		Xylanase		β-glucosidase		Protein	
	Substrate	T(°C)	pH	Pulse	FPase/mL	p (h)	IU/mL	p (h)	IU/mL	p (h)	mg/mL	p(h)
#1	SCBSE	45	5	96	0.10 ± 0.01	72	3.88 ± 0.12	72	0.37 ± 0.04	120	0.06 ± 0.02	120
#2	SCBSE	45	5	48/96	0.13 ± 0.03	120	1.93 ± 0.13	96	0.30 ± 0.06	96	0.08 ± 0.02	96
#3	SCBSE	45	6	48/96	0.10 ± 0.02	96	1.80 ± 0.03	48	0.48 ± 0.03	120	0.10 ± 0.04	96
#4	SCBSE	38–29	6	96	0.18 ± 0.03	120	2.00 ± 0.01	120	0.44 ± 0.02	96	0.17 ± 0.01	120
#5	SCBSE + SUC	45	6	None	0.21 ± 0.02	24	2.70 ± 0.07	48	0.72 ± 0.01	120	0.16 ± 0.05	24
#6	WB	45	6	None	0.06 ± 0.01	24	2.88 ± 0.01	24	1.64 ± 0.10	96	0.13 ± 0.01	24

*SCBSE* steam-exploded sugar cane bagasse;* WB* wheat bran;* SUC* sucrose;* T* temperature; (p), peak activity

**Fig. 2:**
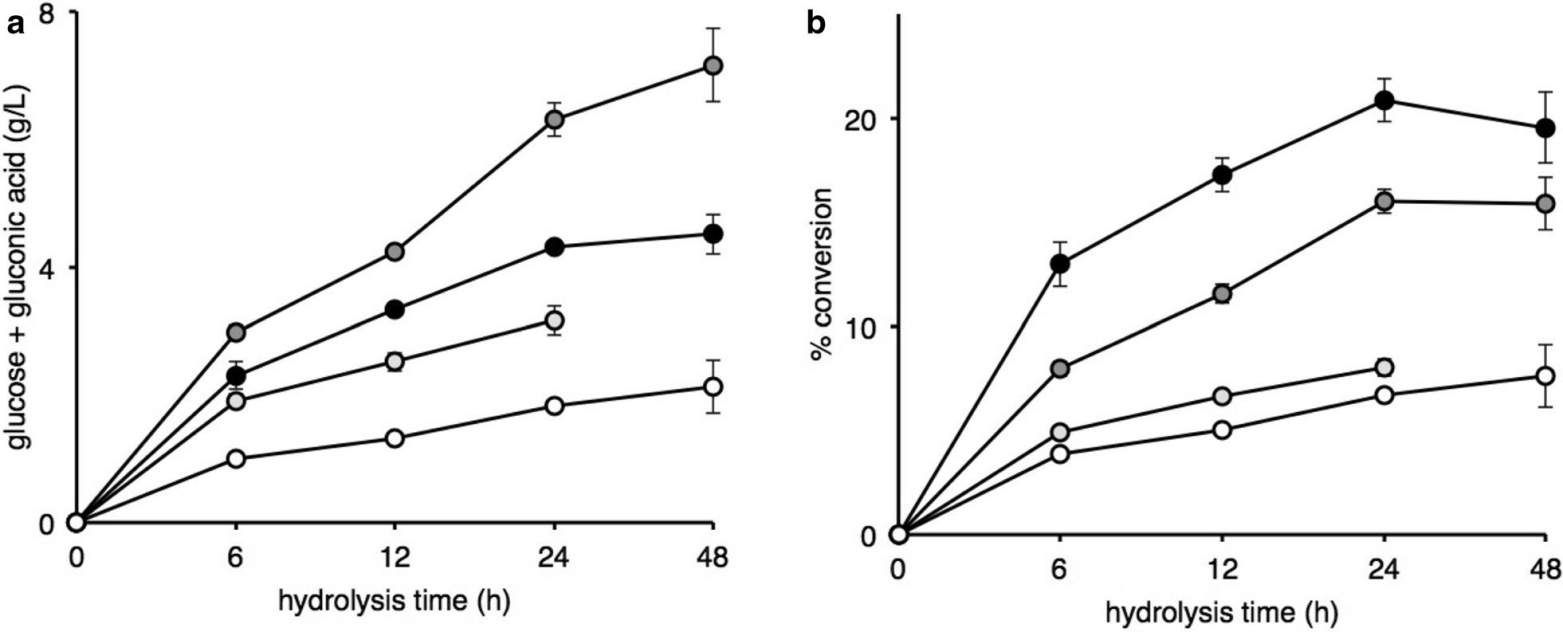
Conversion of various forms of sugar cane bagasse and crystalline cellulose into glucose, gluconic acid, cellobiose and cellobionic acid by *M. thermophila* M77 enzymes. *M. thermophila* M77 secretome produced on “in natura” sugar cane bagasse was concentrated and used to hydrolyze SCBIN (*closed symbols*), EC (cellufloc,* dark shaded* symbols), SCBDL (*light shaded symbols*) and SCBSE (±) at 50 °C. In **a** the release of glucose and gluconic acid is shown and in **b** the conversion percentage from total available sugars was estimated

Figure 2 describes enzymatic biomass hydrolysis into sugars and corresponding aldonic acids of various forms of sugar cane bagasse, “in natura” (SCBIN), delignified (SCBDL), steam-exploded (SCBSE) and purified cellulose (EC) by a crude enzymatic cocktail from *M. thermophila* M77 produced in a bioreactor with SCBIN as the carbon source. After 24 h incubation period, 6.31 g/L of glucose and gluconic acid was produced from cellulose (EC) and 4.31, 3.16 and 1.83 g/L from SCBIN, SCBDL and SCBSE, respectively (Fig. 2a). When the conversion potential of each carbon source was considered a 19.53% conversion was determined for SCBIN and 15.89, 8.02 and 7.63% conversion for EC, SCBDL and SCBSE, respectively (Fig. 2b).

### Secretome protein composition

The secretome (all extracellular non-anchored proteins) produced by *M. thermophila* M77 grown in various carbon sources; SCBIN, SCBDL and SCBSE as well as purified cellulose (avicel and carboxymethylcellulose) and hemicelluloses (xylans, arabinan and arabinoxylan) were determined through LC–MS/MS (Additional file 1: Table S1; Figs. 4, 5). Total extracellular proteins (secretomes) were collected, concentrated by ultra-filtration (10 kDa cutoff), separated by SDS-PAGE, digested with trypsin, subjected to LC–MS/MS and peptides assigned through Mascot and Scaffold to *M. thermophila* ATCC_42464 predicted proteins. In total, 172 proteins were unambiguously identified with positive matching of 21,766 unique peptides (4019 SCBIN, 3661 SCBDL, 4269 SCBSE 3466 celluloses and 4716 hemicelluloses). The spectral counts from two independent experiments were normalized and duplicates averaged in order to enable quantitative comparisons between samples (see Additional file 1: Table S1).

**Fig. 3:**
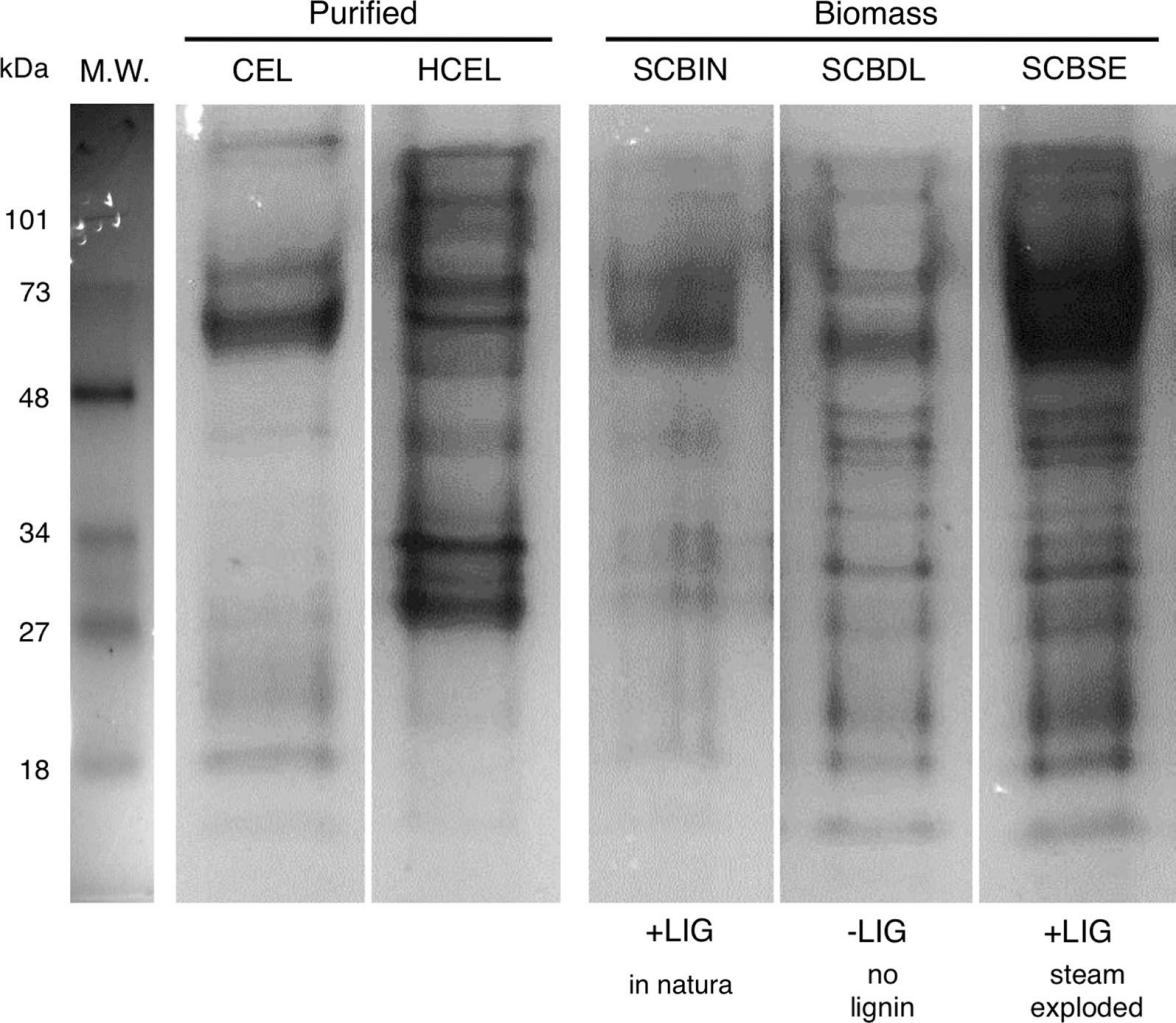
*Myceliophthora thermophila* M77 secretome composition. *M. thermophila* M77 secretomes developed on a variety of carbon sources; sugar cane bagasse (SCB) in natura (SCBIN), delignified (SCBDL) and steam-exploded (SCBSE) as well as purified cellulose (CEL) and hemicellulose (HCEL) were determined through LC–MS/MS from SDS-PAGE separated proteins. 172 proteins were identified with positive matching of 21,766 unique peptides (4019 SCBIN, 3661 SCBDL, 4269 SCBSE 3466 celluloses and 4716 hemicelluloses). CEL, purified cellulose mixture containing 0.5% of avicel and 0.5% carboxymethylcellulose; HCEL, purified hemicellulose mixture containing 0.2% of each; birchwood-, beechwood-, oat spelt-xylan, arabinan and arabinoxylan; SCBIN, milled “in natura” sugar cane bagasse; SCBDL, steam exploded and delignified with sodium hydroxide milled sugar cane bagasse and SCBSE, steam exploded milled sugar cane bagasse. +LIG, lignin present; -LIG, lignin absent; M.W., molecular weight markers shown in kDa

**Fig. 4:**
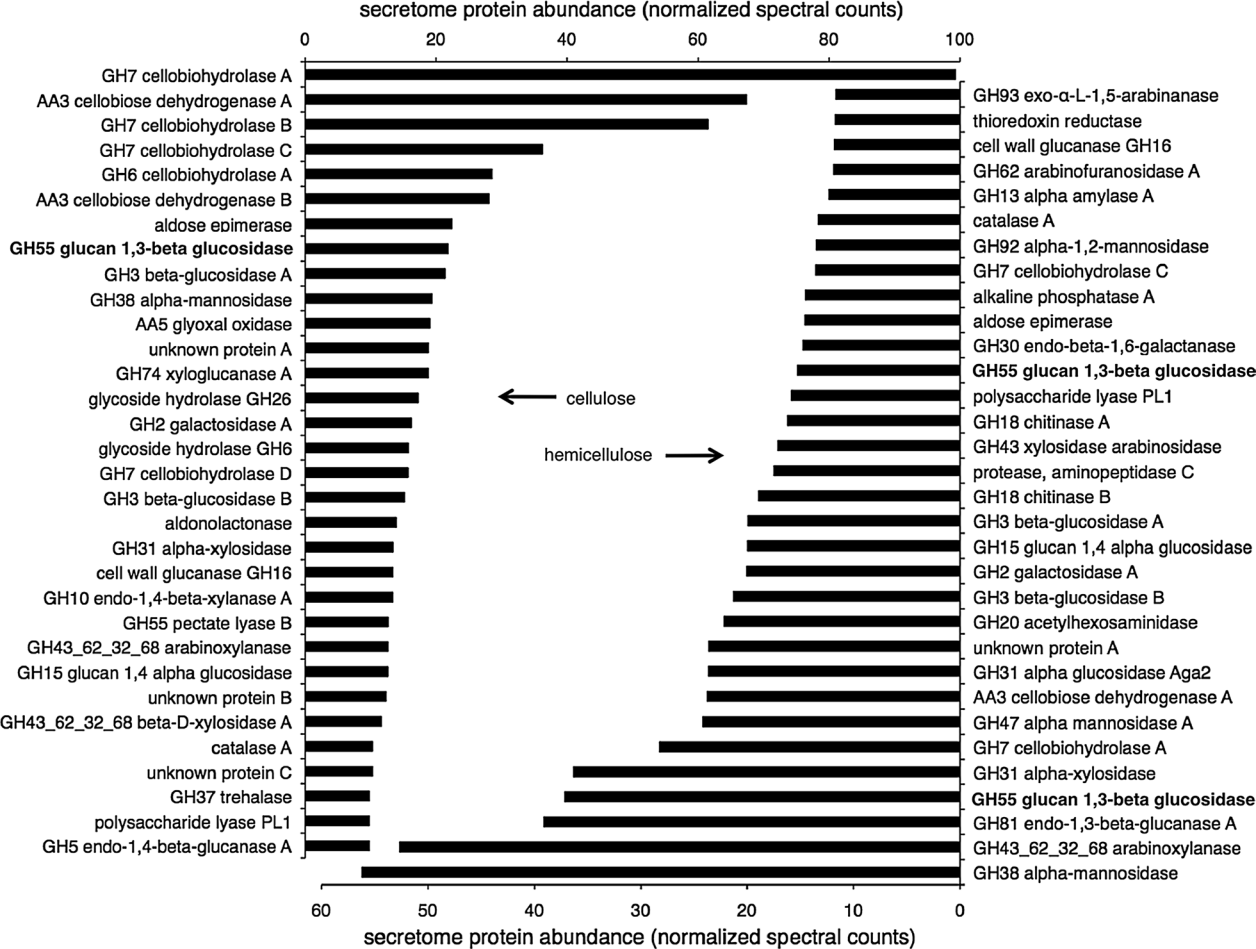
Biomass secretomes. *M. thermophila* M77 secretomes produced on cellulose (*left panel*), a mixture of 0,5% avicel and 0.5% carboxymethylcellulose, or hemicellulose (*right panel*), a mixture of 0.2% of each of three types of xylan, arabinan and arabinoxylan (see “Materials and methods” section) were analyzed by LC–MS/MS and protein abundance reported as normalized spectral counts. A detailed list of annotated protein names along with spectral and quantitative measurement data can be found in Additional file 1: Table S1

Figure 3 shows SDS-PAGE protein profiles of enzymes secreted to the medium as a response to sugar cane bagasse, purified cellulose and hemicellulose and Fig. 4 displays secretome protein abundance profiles of *M. thermophila* M77 grown with purified cellulose (left panel) and a mixture of purified hemicelluloses (right panel). All major proteins in hemicellulose were associated with hemicellulose and pectin breakdown while in cellulose all major proteins were related to cellulose hydrolysis or oxidation.

In conditions where cellulose was the sole carbon source (Fig. 4, left panel) the most abundant proteins in the secretome were: GH7 cellobiohydrolase A (~10% of secretome), AA3 cellobiose dehydrogenase A (~7% of secretome), GH7 cellobiohydrolase B and C (~6 and ~4% of secretome, respectively), GH6 cellobiohydrolase A and AA3 cellobiose dehydrogenase B (~3% of each).

**Fig. 5:**
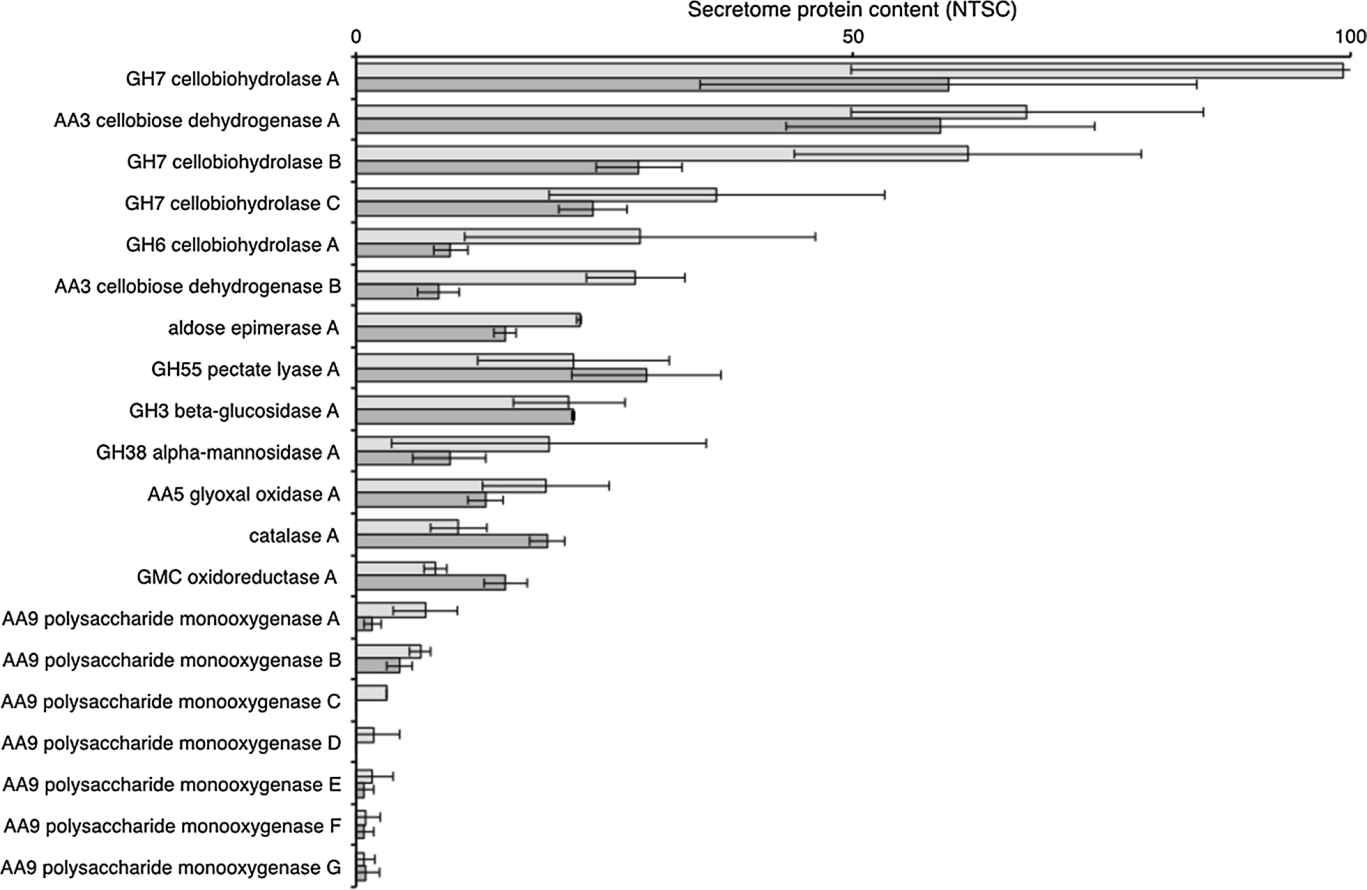
Core cellulose secretome. *M. thermophila* M77 secretomes produced on purified cellulose (CEL, *light shaded bars*) and sugar cane bagasse (SCBIN, *dark shaded bars*). Total extracellular protein abundance was determined by LC–MS/MS and reported as normalized spectral counts. Each bar indicates the normalized spectral counts of one enzyme and abundance ranking was done for SCBIN. Seven AA9 lytic polysaccharide monooxygenases did not rank at the top of any of the studied biomass sources however were added to the present table. A detailed list of annotated protein names along with spectral and quantitative measurement data can be found in Additional file 1: Table S1

When *M. thermophila* M77 was grown in SCBIN, GH7 cellobiohydrolase A and AA3 cellobiose dehydrogenase A were the most abundant proteins both contributing with about 6% of the secretome each (Fig. 5), while other proteins such as GH55 β-1,3-glucanase, GH7 cellobiohydrolase B and C, GH81 endo-1,3-β-glucanase, GH74 xyloglucanase, GH43_62_32_68 arabinoxylanase, GH31 α-xylosidase, GH3 β-glucosidase A, catalase, hypothetical protein (MYCTH_2307339) and GH18 chitinase A contributed with less than 3% of secretome, each. Polysaccharide monooxygenases (seven in total) contributed with less than 1% each (Fig. 5). AA3 cellobiose dehydrogenase A (CdhA) on the other hand was the second most abundant protein (Fig. 4), accounting for about 6% of the secretome protein content.

We thus decided to concisely define the core cellulose secretome (Fig. 5) and further corroborate gene/protein complements. Typically, besides the classical cellobiohydrolase β-glucosidase set of proteins, cellobiose dehydrogenase, a glyoxal oxidase and an unknown GMC oxidoreductase make up the core cellulose secretome (Fig. 5).

Cellulose hydrolases such as cellobiohydrolase A, B, C and D, GH74 xyloglucanase, GH3 β-glucosidase and GH81 endo-β-1,3 glucanase did not adjust in abundance between the three types of biomass. In addition, hemicellulose hydrolases such as GH55 β-1,3-glucanase, GH43_62_32_68 arabinoxylanase, GH7 α-mannosidase and GH2 β-galactosidase also did not vary significantly in abundance among those three biomass substrates.

**Fig. 6:**
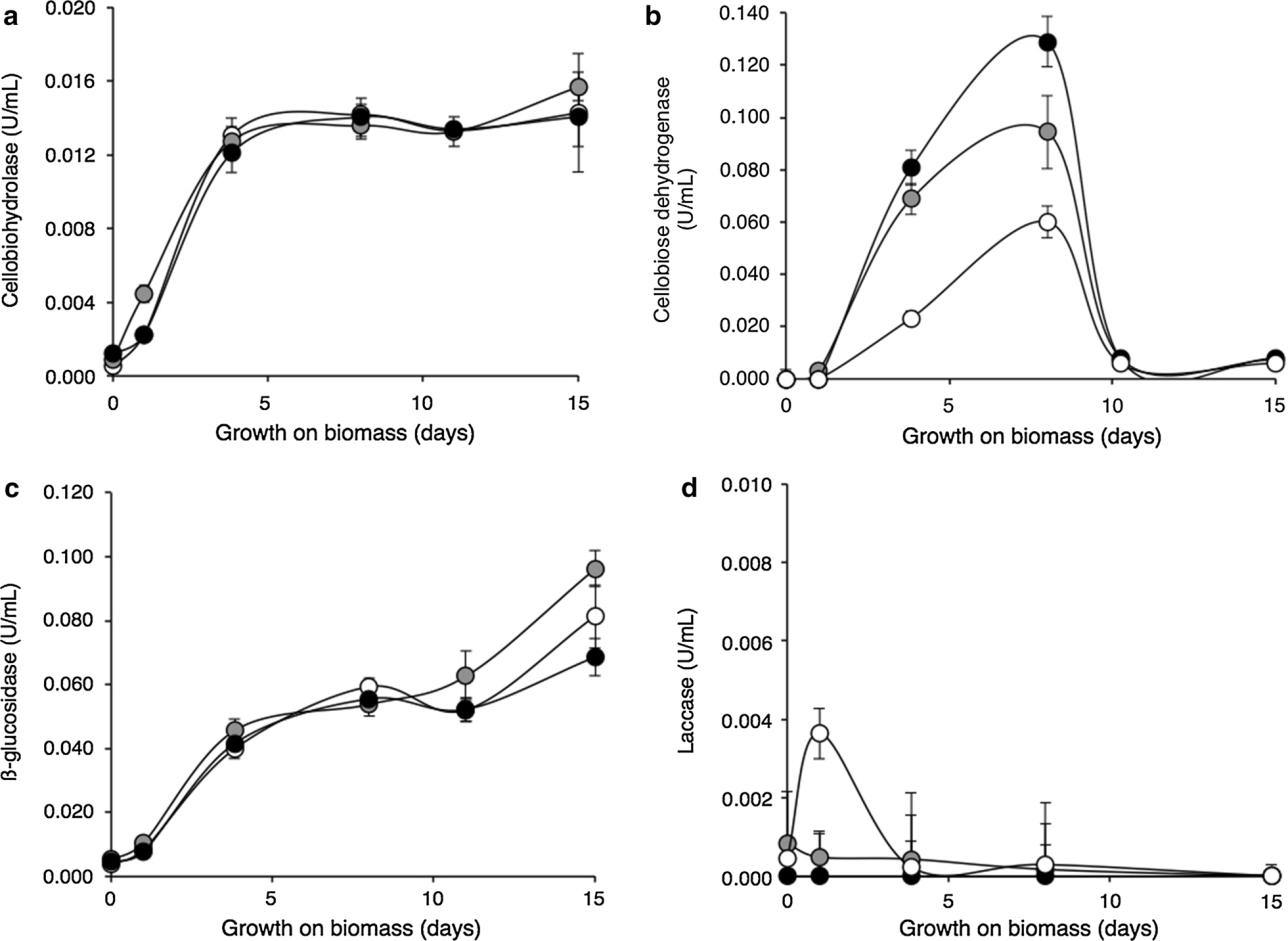
Major enzyme activities on solid biomass degradation. Cellobiohydrolase (**a**) and β-glucosidase (**c**) are the major hydrolases and cellobiose dehydrogenase (**b**) the major oxidase secreted by *M. thermophila* during growth on SCBIN (*closed circles*) “in natura” as well as SCBDL (*shaded circles*) delignified and SCBSE (*open circles*) “steam exploded” biomass. Other oxidative enzymes laccase (**d**), lignin peroxidase (not shown) and Mn-peroxidase (not shown) were detected at very low levels

Figure 6 shows robust cellobiohydrolase, β-glucosidase and cellobiose dehydrogenase activity presence in the fluid of cultures grown on various forms of sugar cane bagasse (SCB). While cellobiohydrolase activity was present at similar levels in all biomass sources accumulating over a 4-day period and remained steady for up to 15 days cellobiose dehydrogenase accumulated for 9 days at differentiated levels in various forms of biomass and then sharply decreased and disappeared from the biomass cultures. β-glucosidase activity followed cellobiohydrolase with no difference in various biomass sources and steady accumulation over time. Laccase however poorly accumulated at the early stages of growth and then disappeared.

## Discussion

Enzymatic cocktails are typically evaluated by their biomass conversion ability, which is specified by the types of enzymes involved in cellulose breakdown producing sugars (glucose and cellobiose) and their respective aldonic acids. Experiments aimed at the production of cellulases using sugar cane bagasse as the carbon source yielded enzyme cocktails with little FPase activity even though the fungus aggressively digested the food source (Figs. 1, 2; Tables 1, 2).

We designed a series of bioreactor experiments (Table 2) to overcome process limitations. With the exception of the presence of sucrose (Table 2, run #5) that doubled the amount of cellulase, none of the other variations seemed to enhance filter paper activity. Other fungal systems such as *Aspergillus nidulans* and *Phanerochaete chrysosporium* produce similar low levels of cellulases and xylanase when growing on solid sorghum stover (Ray et al. 2012; Saykhedkar et al. 2012).

We than designed a biomass hydrolysis experiment using the *M. thermophila* M77 enzymatic cocktail and determined the release of glucose and corresponding gluconic acid. Nevertheless, when the conversion potential of each carbon source was considered, 19.53% for SCBIN and 15.89, 8.02 and 7.63% conversion for EC, SCBDL and SCBSE was observed, respectively (Fig. 2b). However, considering our experiment, the highest cellulase activity detected (0.23 FPU/mL) was applied on SCBIN 5% (w/v) representing an enzyme loading of only ~5 FPU/g of glucan in the hydrolysis experiment, making the observed 20% conversion rate unjustifiable, since other authors have loaded higher filter paper units (FPU) to get similar conversion rates (Adsul et al. 2005; da Silva et al. 2010; Ishihama et al. 2005; Pietrobon et al. 2011; Visser et al. 2015).

Measuring cellulose degradation by methods that only detect hydrolysis mechanisms (for instance FPase activity) may not be sufficient to evaluate the cellulose breakdown power of these enzyme mixtures because the fungus may secrete enzymes that instead of hydrolyzing cellulose, oxidize glycosidic linkages instead.

Secretome protein abundance profiles of *M. thermophila* M77 grown with purified cellulose (Fig. 4, left panel) and a mixture of purified hemicelluloses (Fig. 4, right panel) were constructed. Secretome protein profiles were substrate specific reflecting the nature of the substrate. All major proteins in hemicellulose were associated with hemicellulose and pectin breakdown while in cellulose all major proteins were related to cellulose hydrolysis or oxidation.

GH7 cellobiohydrolases (CbhA) act at the reducing end of a single cellulose chain and CbhA is the only major GH7 cellobiohydrolase that contains a cellulose-binding domain (CBM1). The presence of cellobiohydrolases devoid of cellulose binding domains, CbhB and CbhC in *M. thermophila* M77 secretomes followed similar observations made in other fungi suggesting that these CBM-devoid enzymes collaborate with CBM-bearing exo enzymes on cellulosic chains that have already been pulled apart from the crystalline fiber (Segato et al. 2012).

When grown on SCBIN, GH7 cellobiohydrolase (CbhA) and AA3 cellobiose dehydrogenase (CdhA) were the most abundant proteins both contributing with about 6% of the secretome each (Fig. 5), while other proteins contributed with less than 3% of secretome, each.

The *M. thermophila* M77 CdhA is a complete cellobiose dehydrogenase, a flavin-dependent dehydrogenase connected through a flexible linker to a heme-binding cytochrome and a true cellulose-binding domain (CBM1) (Tan et al. 2015). CdhA generates electrons by oxidation of cellobiose (perhaps generated by CbhA) and longer cellodextrins to 1-5-δ-lactones (Westermark and Eriksson 1975). Lactones hydrolyze spontaneously in solution, or enzymatically by lactonases (also present in the secretome), to generate aldonic acids (Beeson et al. 2011). Electrons generated by the flavin-dependent dehydrogenase are shuttled via heme-binding cytochrome to the recently discovered copper dependent polysaccharide monooxygenases (PMO’s) that in turn oxidize glycoside bonds in crystalline cellulose, hemicellulose and pectin (Beeson et al. 2011; Canevascini et al. 1991).

Remarkably, Fig. 5 shows that when the fungus grew in biomass (SCBIN) or purified cellulose (CEL) AA3 cellobiose dehydrogenase A was present in about the same concentration (about 50%) while GH7 and GH6 cellobiohydrolases were present at higher levels in CEL. Thus, the CdhA mediated electrons could be transferred to a wide range of oxygenases (Hemsworth et al. 2013; Westermark and Eriksson 1975; Zamocky et al. 2006). Other enzymes such as GH31 α-xylosidase, GH18 chitinase A (but not chitinase B), GMC oxidoreductase (an unknown oxidoreductase), GH43 xylosidase arabinosidase and AA5 glyoxal oxidase, followed a similar pattern, low abundance in delignified biomass and abundant in whole forms of biomass. The function of GMC oxidoreductase (GloA), glyoxal oxidase (GoxA) and GH18 chitinase even though clearly annotated by bioinformatics remain unclear and undefined.

Thus, based on the protein profile of secreted proteins and enzymatic activity detected it appears that the fungus accesses all available polymers, cellulose hemicellulose-pectin and lignin but does not produce hydrolysis products exclusively.

The participation of oxidation reactions coupled to lignin decomposition in the breakdown of cellulose chains (Beeson et al. 2012; Phillips et al. 2011), may explain the discrepancy observed between absolute FPase activity values in bioreactor experiments and the real (total) power of cellulose breakdown observed in biomass hydrolysis experiments. Furthermore, it is possible that CdhA, GloA and GoxA fail to interact and transfer electrons to cellulose, lignin and hemicellulose acceptor proteins, they generate an excess of hydrogen peroxide, which may directly oxidize glycosidic and phenolic bonds by a currently unknown mechanism.

*Myceliophthora thermophila* M77 produces specific secretomes that mirror the cell wall composition, formulate a mixed set of enzymes that in addition to hydrolyze glycoside bonds also promote coupled oxidation of cellulose and other biomass components enhancing the overall biomass degradation process. The secretome protein signature of *M. thermophila* M77 revealed cellobiose dehydrogenase (21% of the total secretome) as the major player in cellulose oxidation partnering perhaps with oxidation proteins such as glyoxal oxidase (4% of the secretome content) or glucose oxidase (not very abundant). The exact function of these enzymes remains uncertain.

## Additional file

**Additional file 1.** Comprehensive LC–MS/MS secretome analysis.

## Abbreviations

AA3: auxiliary activities 3
ABTS: 2,2′-Azino-bis(3-ethylbenzothiazoline-6-sulfonic acid) diammonium salt
CBM: carbohydrate binding domain
DCPIP: 2,6-dichlorophenol-indophenol
DNS: dinitrosalicylic acid
EC: commercial microcrystalline cellulose
FDR: false discovery rate
FOS: fructoligosaccharides
FPase: filter paper cellulase activity
FPU: filter paper unit
HPLC: high performance liquid chromatography
IU: enzyme unit
GH3 7, 18, 74, 81: glycoside hydrolase family 3, 7,18, 74 and 81
GLY: glycerol
LAC: lactose
LC–MS/MS: liquid chromatography–tandem mass spectrometry
NCBI: National Center for Biotechnology Information (NIH, US)
NTSC: normalized total spectral counts
PMO: polysaccharide monoxygenase
pNPC: *p*-nitrophenol-β-d-cellobioside
pNPG: *p*-nitrophenol-β-d-glucopyranoside
SCB: sugar cane bagasse
SCBDL: lignin removed (sodium hydroxide extracted) and steam exploded sugar cane bagasse
SCBIN: in natura” milled (200-μm particle size) sugar cane bagasse
SCBSE: steam exploded sugar cane bagasse
SDS-PAGE: SDS polyacrylamide gel electrophoresis
SM: milled soybeans
SUC: sucrose
TSC: total spectral counts
WB: wheat bran
