# Supplementary material for: Myceliophthora thermophila M77 utilizes hydrolytic and oxidative mechanisms to deconstruct biomass
Source: AMB Express. 2016 Nov 2;6:103. doi: 10.1186/s13568-016-0276-y (PMC5093097; doi:10.1186/s13568-016-0276-y)
Supplement: Supplementary file 1 — Additional file 1. Comprehensive LC–MS/MS secretome analysis. [file 13568_2016_276_MOESM1_ESM.pdf]

# *Myceliophthora thermophila* M77 utilizes alternate glycosidic bond breaking mechanisms to deconstruct biomass

Hévila Brognaro dos Santos 1, 3, 5, Thaís Milena Souza Bezerra 4, 5, José G. C. Pradella 3, Priscila Delabona 3, Deise Lima 3, Eleni Gomes 2, Steve D. Hartson6, Janet Rogers6, Brian Couger 5 and Rolf Prade 5, *

1 Instituto de Física de São Carlos, Universidade de São Paulo, São Carlos/SP, 2 Laboratório de Microbiologia e Bioquímica Aplicada, Departamento de Biologia, IBILCE/UNESP, Rua Cristovão Colombo, 2265 Bairro Jd. Nazareth, CEP 15054-000 São José do Rio Preto-SP/Brazil. 3 Laboratório Nacional de Ciência e Tecnologia do Bioetanol, Rua Giuseppe Máximo Scolfaro, 10.000, Bairro Guará, CEP 13083-970, Campinas-SP/Brasil. 4 Laboratório de Enzimologia, Instituto de Química, UNESP, Araraquara São Paulo. 5Departments of Microbiology & Molecular Genetics and 6Biochemistry & Molecular Biology, Oklahoma State University, Stillwater, OK 74078, USA.
AMB Express

Running title: *M. thermophila* Biomass Degradation by Oxidation

* Corresponding author: R. Prade Email: prade@okstate.edu, Tel: 1-405-744-7522, Fax: 1-405-744-67

**Table S1** Comprehensive LC-MS/MS secretome analysis

**CEL (C)**, 0.5% Avicel, 0.5% carboxymethylcellulose; **HCE (H)**, 0.2% of each oat spelt-, birchwood-, beech wood- xylan, arabinan, arabinoxylan; **SCBIN (I)**, "*in natura*" sugar cane bagasse; **SCBDL(D)**, sugar cane bagasse lignin removed; **SCBSE (S)**, sugar cane bagasse "steam exploded"; **HB1**, sample 1; **HB2**, sample 2; **TSC**, total spectral counts, **N-TSC**, normalized TSC; **AVE**, HB1 and H23 averaged NTSC.

| obs | Structural features: Domains cat 1 | Domains cat 2 | binding | Protein: functional name (164) | locus tag | locus name | Organism source | Accession GI | M.W. kDa | CEL HB1 TSC | CEL HB1 N-TSC | CEL HB2 TSC | CEL HB2 N-TSC | CEL AVE | HCE (H) HB1 TSC | HCE HB1 N-TSC | HCE HB2 TSC | HCE HB2 N-TSC | HCE AVE | SCBIN (I) HB1 TSC | SCBIN HB1 N-TSC | SCBIN HB2 TSC | SCBIN HB2 N-TSC | SCBIN AVE | SCBDL (D) HB1 TSC | SCBDL HB1 N-TSC | SCBDL HB2 TSC | SCBDL HB2 N-TSC | SCBDL AVE | SCBSE (S) HB1 TSC | SCBSE HB1 N-TSC | SCBSE HB2 TSC | SCBSE HB2 N-TSC | SCBSE AVE |
|---|---|---|---|---|---|---|---|---|---|---|---|---|---|---|---|---|---|---|---|---|---|---|---|---|---|---|---|---|---|---|---|---|---|---|
| Top tier of secreted proteins (Top 75% of N-TSC in each condition) | | | | | | | | | | | | | | | | | | | | | | | | | | | | | | | | | | |
| | DOMON | GMC_oxred_c | CBM1 | AA3 cellobiose dehydrogenase A | MYCTH_111388 | CdhAA3A | *Myceliophthora thermophila ATCC 42464* | 3319315 | 88 | 106 | 55 | 78 | 80 | 67 | 61 | 26 | 35 | 22 | 24 | 98 | 48 | 93 | 70 | 59 | 48 | 27 | 42 | 34 | 30 | 127 | 59 | 115 | 76 | 67 |
| | DOMON | GMC_oxred_c | - | AA3 cellobiose dehydrogenase B | MYCTH_81925 | CdhAA3B | *Myceliophthora thermophila ATCC 42464* | 347011813 | 84 | 61 | 32 | 24 | 25 | 28 | 14 | 6 | 4 | 2 | 4 | 20 | 10 | 9 | 7 | 8 | 14 | 8 | 10 | 8 | 8 | 30 | 14 | 10 | 7 | 10 |
| | E-set | AA_5 | 4xWSC | AA5 glyoxal oxidase | MYCTH_2294895 | GoxAA5A | *Myceliophthora thermophila ATCC 42464* | 347006010 | 105 | 28 | 15 | 23 | 24 | 19 | 16 | 7 | 18 | 11 | 9 | 24 | 12 | 19 | 14 | 13 | 12 | 7 | 14 | 11 | 9 | 29 | 13 | 18 | 12 | 13 |
| | - | GH_61 | - | AA9 lytic polysaccharide monooxygenase A | MYCTH_80312 | LpmoAA9A | *Myceliophthora thermophila ATCC 42464* | 347010683 | 32 | 9 | 5 | 9 | 9 | 7 | 0 | 0 | 0 | 0 | 0 | 2 | 1 | 3 | 2 | 2 | 0 | 0 | 0 | 0 | 0 | 0 | 0 | 0 | 0 | 0 |
| | - | GH_61 | CBM1 | AA9 lytic polysaccharide monooxygenase B | MYCTH_46583 | LpmoAA9B | *Myceliophthora thermophila ATCC 42464* | 347009055 | 35 | 11 | 6 | 7 | 7 | 6 | 3 | 1 | 0 | 0 | 1 | 7 | 3 | 7 | 5 | 4 | 4 | 2 | 0 | 0 | 1 | 10 | 5 | 5 | 3 | 4 |
| | - | ? | - | aldonolactonase | MYCTH_2298860 | AolA | *Myceliophthora thermophila* | 313667052 | 43 | 32 | 17 | 11 | 11 | 14 | 12 | 5 | 10 | 6 | 6 | 13 | 6 | 11 | 8 | 7 | 33 | 18 | 14 | 11 | 15 | 23 | 11 | 21 | 14 | 12 |
| | - | ? | - | aldose epimerase A | MYCTH_103702 | AleA | *Myceliophthora thermophila ATCC 42464* | 347012126 | 41 | 43 | 22 | 22 | 23 | 22 | 31 | 13 | 26 | 16 | 15 | 29 | 14 | 21 | 16 | 15 | 56 | 31 | 35 | 28 | 30 | 39 | 18 | 35 | 23 | 21 |
| | - | alkPPc | - | alkaline phosphatase A | MYCTH_2304549 | PhoA | *Myceliophthora thermophila ATCC 42464* | 347010366 | 66 | 3 | 2 | 0 | 0 | 1 | 38 | 16 | 21 | 13 | 15 | 14 | 7 | 5 | 4 | 5 | 10 | 6 | 0 | 0 | 3 | 17 | 8 | 0 | 0 | 4 |
| | - | Ntn_aspariganse | - | asparaginase | | | *Myceliophthora thermophila ATCC 42464* | 347012213 | 41 | 4 | 2 | 0 | 0 | 1 | 10 | 4 | 6 | 4 | 4 | 9 | 4 | 7 | 5 | 5 | 10 | 6 | 10 | 8 | 7 | 5 | 2 | 8 | 5 | 4 |
| | | | | catalase A | MYCTH_80916 | CatA | *Myceliophthora thermophila ATCC 42464* | 347010301 | 78 | 16 | 8 | 12 | 12 | 10 | 25 | 11 | 26 | 16 | 13 | 42 | 20 | 24 | 18 | 19 | 48 | 27 | 30 | 24 | 25 | 33 | 15 | 29 | 19 | 17 |
| | | | | CE8 pectinesterase | MYCTH_39035 | PecCE8A | *Myceliophthora thermophila ATCC 42464* | 347013279 | 36 | 2 | 1 | 0 | 0 | 1 | 23 | 10 | 21 | 13 | 11 | 13 | 6 | 13 | 10 | 8 | 0 | 0 | 0 | 0 | 0 | 0 | 0 | 0 | 0 | 0 |
| | - | GH_10 | CBM1 | GH10 endo-1,4-beta-xylanase A | MYCTH_112050 | XynGH10A | *Myceliophthora thermophila ATCC 42464* | 347012973 | 45 | 22 | 11 | 15 | 15 | 13 | 14 | 6 | 11 | 7 | 6 | 16 | 8 | 12 | 9 | 8 | 9 | 5 | 9 | 7 | 6 | 11 | 5 | 11 | 7 | 6 |
| | | | | GH114 glycoside hydrolase | | | *Myceliophthora thermophila ATCC 42464* | 347012343 | 39 | 17 | 9 | 10 | 10 | 10 | 14 | 6 | 7 | 4 | 5 | 0 | 0 | 0 | 0 | 0 | 4 | 2 | 0 | 0 | 1 | 16 | 7 | 14 | 9 | 8 |
| | - | AmyAc | DUF1966 | GH13 alpha amylase A | MYCTH_2305783 | AmyGH13A | *Myceliophthora thermophila ATCC 42464* | 347010961 | 58 | 8 | 4 | 0 | 0 | 2 | 29 | 12 | 20 | 12 | 12 | 16 | 8 | 6 | 4 | 6 | 9 | 5 | 4 | 3 | 4 | 13 | 6 | 7 | 5 | 5 |
| | - | GH_15 | CBM20 | GH15 glucan 1,4 alpha glucosidase | MYCTH_72393 | AgaGH15A | *Myceliophthora thermophila ATCC 42464* | 347014101 | 67 | 27 | 14 | 11 | 11 | 13 | 36 | 15 | 40 | 25 | 20 | 23 | 11 | 18 | 13 | 12 | 38 | 21 | 30 | 24 | 23 | 37 | 17 | 35 | 23 | 20 |
| | - | ? | - | GH16 cell wall glucanase | MYCTH_2315557 | | *Myceliophthora thermophila ATCC 42464* | 347011420 | 41 | 22 | 11 | 15 | 15 | 13 | 28 | 12 | 19 | 12 | 12 | 26 | 13 | 18 | 13 | 13 | 30 | 17 | 21 | 17 | 17 | 26 | 12 | 22 | 14 | 13 |
| | | | | GH16 glycoside hydrolase | MYCTH_48804 | GH16B | *Myceliophthora thermophila ATCC 42464* | 347010669 | 24 | 11 | 6 | 8 | 8 | 7 | 5 | 2 | 0 | 0 | 1 | 7 | 3 | 8 | 6 | 5 | 14 | 8 | 9 | 7 | 7 | 10 | 5 | 10 | 7 | 6 |
| | | | | GH17 glycoside hydrolase | MYCTH_2315007 | GH17 | *Myceliophthora thermophila ATCC 42464* | 347009838 | 43 | 8 | 4 | 4 | 4 | 4 | 13 | 6 | 11 | 7 | 6 | 11 | 5 | 5 | 4 | 5 | 19 | 11 | 12 | 10 | 10 | 11 | 5 | 11 | 7 | 6 |
| | - | GH18 | - | GH18 chitinase A | MYCTH_2308241 | ChiGH18A | *Myceliophthora thermophila ATCC 42464* | 347012251 | 45 | 18 | 9 | 0 | 0 | 5 | 56 | 24 | 14 | 9 | 16 | 45 | 22 | 18 | 13 | 18 | 13 | 7 | 7 | 6 | 6 | 38 | 18 | 10 | 7 | 12 |
| | - | GH_18 | - | GH18 chitinase B | MYCTH_50608 | ChiGH18B | *Myceliophthora thermophila ATCC 42464* | 347010813 | 46 | 19 | 10 | 7 | 7 | 9 | 34 | 14 | 38 | 23 | 19 | 23 | 11 | 16 | 12 | 12 | 19 | 11 | 13 | 10 | 10 | 24 | 11 | 23 | 15 | 13 |
| | - | GH_2 | - | GH2 galactosidase A | MYCTH_114497 | GalGH2A | *Myceliophthora thermophila ATCC 42464* | 347007020 | 66 | 29 | 15 | 17 | 17 | 16 | 42 | 18 | 36 | 22 | 20 | 29 | 14 | 25 | 19 | 16 | 33 | 18 | 21 | 17 | 18 | 40 | 19 | 29 | 19 | 19 |
| | - | GH_20 | - | GH20 acetylhexosaminidase | MYCTH_2294755 | | *Myceliophthora thermophila ATCC 42464* | 347005947 | 65 | 11 | 6 | 11 | 11 | 8 | 42 | 18 | 43 | 26 | 22 | 25 | 12 | 18 | 13 | 13 | 26 | 14 | 22 | 18 | 16 | 18 | 8 | 24 | 16 | 12 |
| | - | GH_26 | CBM6 | GH26 glycoside hydrolase | MYCTH_99077 | GH26 | *Myceliophthora thermophila ATCC 42464* | 347006182 | 53 | 37 | 19 | 15 | 15 | 17 | 13 | 6 | 5 | 3 | 4 | 20 | 10 | 12 | 9 | 9 | 14 | 8 | 10 | 8 | 8 | 31 | 14 | 12 | 8 | 11 |
| | - | GH_3 | Fn3 | GH3 beta-glucosidase A | MYCTH_66804 | GluGH3A | *Myceliophthora thermophila ATCC 42464* | 347010857 | 78 | 49 | 25 | 17 | 17 | 21 | 43 | 18 | 35 | 22 | 20 | 45 | 22 | 29 | 22 | 22 | 68 | 38 | 40 | 32 | 35 | 66 | 31 | 26 | 17 | 24 |
| | - | GH_3 | Fn3 | GH3 beta-glucosidase B | MYCTH_80304 | GluGH3B | *Myceliophthora thermophila ATCC 42464* | 347010689 | 95 | 23 | 12 | 18 | 18 | 15 | 35 | 15 | 45 | 28 | 21 | 32 | 16 | 18 | 13 | 15 | 40 | 22 | 23 | 18 | 20 | 30 | 14 | 23 | 15 | 15 |
| | | | | GH3 glycoside hydrolase | MYCTH_58882 | GH3A | *Myceliophthora thermophila ATCC 42464* | 347012994 | 84 | 10 | 5 | 5 | 5 | 5 | 26 | 11 | 6 | 4 | 7 | 17 | 8 | 5 | 4 | 6 | 10 | 6 | 0 | 0 | 3 | 8 | 4 | 5 | 3 | 4 |
| | - | ? | - | GH30 endo-beta-1,6-galactanase | MYCTH_2301720 | BlglGH30A | *Myceliophthora thermophila ATCC 42464* | 347009128 | 53 | 11 | 6 | 12 | 12 | 9 | 29 | 12 | 28 | 17 | 15 | 29 | 14 | 21 | 16 | 15 | 18 | 10 | 15 | 12 | 11 | 13 | 6 | 31 | 20 | 13 |
| | - | PLNO2808 | - | GH31 alpha glucosidase A | MYCTH_2313137 | AgaGH31A | *Myceliophthora thermophila ATCC 42464* | 367036173 | 46 | 18 | 9 | 6 | 6 | 8 | 46 | 20 | 45 | 28 | 24 | 21 | 10 | 21 | 16 | 13 | 19 | 11 | 15 | 12 | 11 | 15 | 7 | 12 | 8 | 7 |
| retaining | - | acid/base | - | GH31 alpha-xylosidase A | MYCTH_2305458 | AxlGH31A | *Myceliophthora thermophila ATCC 42464* | 347010772 | 97 | 40 | 21 | 6 | 6 | 13 | 107 | 46 | 44 | 27 | 36 | 54 | 26 | 24 | 18 | 22 | 30 | 17 | 21 | 17 | 17 | 61 | 28 | 31 | 20 | 24 |
| | - | Treha | - | GH37 trehalase | MYCTH_75490 | | *Myceliophthora thermophila ATCC 42464* | 347005659 | 78 | 20 | 10 | 9 | 9 | 10 | 10 | 4 | 9 | 6 | 5 | 17 | 8 | 10 | 7 | 8 | 7 | 4 | 7 | 6 | 5 | 19 | 9 | 9 | 6 | 7 |
| | - | GH_38 | - | GH38 alpha-mannosidase | MYCTH_2295939 | ManGH38A | *Myceliophthora thermophila ATCC 42464* | 347006480 | 123 | 59 | 31 | 8 | 8 | 19 | 160 | 68 | 72 | 44 | 56 | 14 | 7 | 16 | 12 | 9 | 69 | 38 | 20 | 16 | 27 | 89 | 41 | 48 | 32 | 37 |
| inverting | - | GH43 | - | GH43 beta xylosidase | MYCTH_2303298 | XynGH43A | *Myceliophthora thermophila ATCC 42464* | 347009817 | 61 | 27 | 14 | 11 | 11 | 13 | 90 | 38 | 109 | 67 | 53 | 49 | 24 | 29 | 22 | 23 | 50 | 28 | 26 | 21 | 24 | 36 | 17 | 31 | 20 | 19 |
| | - | | | GH43 galactan 1,3 beta galactosidase | MYCTH_50820 | GalGalGH43A | *Myceliophthora thermophila ATCC 42464* | 347010818 | 49 | 8 | 4 | 5 | 5 | 5 | 26 | 11 | 16 | 10 | 10 | 14 | 7 | 15 | 11 | 9 | 14 | 8 | 11 | 9 | 8 | 13 | 6 | 4 | 3 | 4 |
| | | | | GH43 glycoside hydrolase | MYCTH_103032 | GH43A | *Myceliophthora thermophila ATCC 42464* | 347010937 | 35 | 5 | 3 | 0 | 0 | 1 | 15 | 6 | 9 | 6 | 6 | 9 | 4 | 11 | 8 | 6 | 6 | 3 | 3 | 2 | 3 | 5 | 2 | 8 | 5 | 4 |
| | - | GH_43_62_32_6 | - | GH43 xylosidase arabinosidase A | MYCTH_2301869 | AxlAraGH43A | *Myceliophthora thermophila ATCC 42464* | 347009205 | 67 | 12 | 6 | 4 | 4 | 5 | 40 | 17 | 28 | 17 | 17 | 28 | 14 | 12 | 9 | 11 | 9 | 5 | 6 | 5 | 5 | 19 | 9 | 6 | 4 | 6 |
| | - | ? | - | GH43_62_32_68 beta-D-xylosidase A | MYCTH_2072383 | XldGH43A | *Myceliophthora thermophila ATCC 42464* | 347014190 | 55 | 33 | 17 | 6 | 6 | 12 | 9 | 4 | 4 | 2 | 3 | 37 | 18 | 12 | 9 | 14 | 34 | 19 | 6 | 5 | 12 | 27 | 13 | 8 | 5 | 9 |
| | - | GH_47 | - | GH47 alpha mannosidase A | MYCTH_2139585 | ManGH47A | *Myceliophthora thermophila ATCC 42464* | 347011389 | 58 | 23 | 12 | 3 | 3 | 7 | 50 | 21 | 44 | 27 | 24 | 34 | 17 | 18 | 13 | 15 | 28 | 16 | 18 | 14 | 15 | 24 | 11 | 23 | 15 | 13 |
| | | | | GH5 beta galactanase | | | *Myceliophthora thermophila ATCC 42464* | 347011035 | 47 | 4 | 2 | 0 | 0 | 1 | 16 | 7 | 15 | 9 | 8 | 6 | 3 | 4 | 3 | 3 | 4 | 2 | 0 | 0 | 1 | 0 | 0 | 0 | 0 | 0 |
| | - | GH_5 endo | CBM1 | GH5 endo-1,4-beta-glucanase A | MYCTH_86753 | CelGH5A | *Myceliophthora thermophila ATCC 42464* | 343409229 | 42 | 14 | 7 | 12 | 12 | 10 | 5 | 2 | 4 | 2 | 2 | 6 | 3 | 9 | 7 | 5 | 5 | 3 | 0 | 0 | 1 | 8 | 4 | 9 | 6 | 5 |
| | | | | GH55 glycoside hydrolase | MYCTH_2299780 | GH55 | *Myceliophthora thermophila ATCC 42464* | 347008205 | 95 | 12 | 6 | 6 | 6 | 6 | 13 | 6 | 8 | 5 | 5 | 11 | 5 | 13 | 10 | 8 | 14 | 8 | 10 | 8 | 8 | 14 | 7 | 10 | 7 | 7 |
| | | | | GH55 β-1,3-glucanase A | MYCTH_102522 | BglGH55A | *Myceliophthora thermophila ATCC 42464* | 347011592 | 82 | 29 | 15 | 28 | 29 | 22 | 69 | 29 | 73 | 45 | 37 | 49 | 24 | 46 | 34 | 29 | 42 | 23 | 48 | 38 | 31 | 47 | 22 | 53 | 35 | 28 |
| | | | | GH55 β-1,3-glucanase B | MYCTH_2305407 | BglGH55B | *Myceliophthora thermophila ATCC 42464* | 347010723 | 84 | 33 | 17 | 8 | 8 | 13 | 40 | 17 | 22 | 14 | 15 | 32 | 16 | 20 | 15 | 15 | 43 | 24 | 35 | 28 | 26 | 24 | 11 | 27 | 18 | 14 |
| | - | GH6 | CBM1 | GH6 cellobiohydrolase A | MYCTH_66729 | CbhGH6A | *Myceliophthora thermophila ATCC 42464* | 347008300 | 51 | 31 | 16 | 40 | 41 | 29 | 6 | 3 | 3 | 2 | 2 | 22 | 11 | 11 | 8 | 9 | 23 | 13 | 12 | 10 | 11 | 28 | 13 | 30 | 20 | 16 |
| | - | GH_6 | - | GH6 glycoside hydrolase | MYCTH_2303045 | GH6 | *Myceliophthora thermophila ATCC 42464* | 347009703 | 41 | 41 | 21 | 10 | 10 | 16 | 7 | 3 | 0 | 0 | 1 | 16 | 8 | 10 | 7 | 8 | 0 | 0 | 0 | 0 | 0 | 8 | 4 | 0 | 0 | 2 |
| | - | GH_62 | - | GH62 arabinofuranosidase A | MYCTH_55982 | AraAGH62A | *Myceliophthora thermophila ATCC 42464* | 347012329 | 35 | 14 | 7 | 0 | 0 | 4 | 24 | 10 | 22 | 14 | 12 | 20 | 10 | 14 | 10 | 10 | 10 | 6 | 4 | 3 | 4 | 7 | 3 | 8 | 5 | 4 |
| | - | GH7_CBH_EG | CBM1 | GH7 cellobiohydrolase A | MYCTH_109566 | CbhGH7A | *Myceliophthora thermophila ATCC 42464* | 347008056 | 56 | 124 | 64 | 131 | 134 | 99 | 49 | 21 | 58 | 36 | 28 | 86 | 42 | 103 | 77 | 60 | 92 | 51 | 102 | 82 | 66 | 126 | 59 | 113 | 74 | 67 |
| | - | GH7_CBH_EG | - | GH7 cellobiohydrolase B | MYCTH_97137 | CbhGH7B | *Myceliophthora thermophila ATCC 42464* | 347013780 | 49 | 95 | 49 | 72 | 74 | 62 | 26 | 11 | 15 | 9 | 10 | 52 | 25 | 42 | 31 | 28 | 74 | 41 | 59 | 47 | 44 | 72 | 34 | 55 | 36 | 35 |
| | - | GH7_CBH_EG | - | GH7 cellobiohydrolase C | MYCTH_116157 | CbhGH7C | *Myceliophthora thermophila ATCC 42464* | 347011876 | 49 | 47 | 24 | 47 | 48 | 36 | 32 | 14 | 22 | 14 | 14 | 44 | 21 | 35 | 26 | 24 | 34 | 19 | 42 | 34 | 26 | 49 | 23 | 53 | 35 | 29 |
| | - | GH7_CBH_EG | CBM1 | GH7 cellobiohydrolase D | MYCTH_111372 | CbhGH7D | *Myceliophthora thermophila ATCC 42464* | 347010710 | 49 | 27 | 14 | 17 | 17 | 16 | 13 | 6 | 13 | 8 | 7 | 31 | 15 | 21 | 16 | 15 | 33 | 18 | 40 | 32 | 25 | 38 | 18 | 44 | 29 | 23 |
| | - | GH71_99 | - | GH71 alpha-1,3-glucanase A | MYCTH_140261 | AgnGH71A | *Myceliophthora thermophila ATCC 42464* | 347013742 | 67 | 13 | 7 | 10 | 10 | 8 | 27 | 12 | 17 | 10 | 11 | 16 | 8 | 8 | 6 | 7 | 17 | 9 | 14 | 11 | 10 | 17 | 8 | 16 | 11 | 9 |
| | | | | GH72 glycoside hydrolase | | | *Myceliophthora thermophila ATCC 42464* | 347009194 | 57 | 6 | 3 | 5 | 5 | 4 | 0 | 0 | 0 | 0 | 0 | 4 | 2 | 5 | 4 | 3 | 5 | 3 | 5 | 4 | 3 | 7 | 3 | 12 | 8 | 6 |
| | - | GH_74 | - | GH74 xyloglucanase A | MYCTH_116384 | XlgGH74A | *Myceliophthora thermophila ATCC 42464* | 347011442 | 79 | 41 | 21 | 16 | 16 | 19 | 23 | 10 | 17 | 10 | 10 | 45 | 22 | 33 | 25 | 23 | 46 | 26 | 30 | 24 | 25 | 57 | 27 | 53 | 35 | 31 |
| | - | GH_81 | - | GH81 endo 1,3(4) beta glucanase A | MYCTH_2306588 | CelGH81A | *Myceliophthora thermophila ATCC 42464* | 347011396 | 91 | 28 | 15 | 4 | 4 | 9 | 87 | 37 | 67 | 41 | 39 | 61 | 30 | 34 | 25 | 28 | 68 | 38 | 42 | 34 | 36 | 60 | 28 | 36 | 24 | 26 |
| | - | COG3537 | - | GH92 alpha-1,2-mannosidase A | MYCTH_2298363 | ManGH92A | *Myceliophthora thermophila ATCC 42464* | 347007534 | 90 | 7 | 4 | 5 | 5 | 4 | 36 | 15 | 19 | 12 | 14 | 19 | 9 | 12 | 9 | 9 | 31 | 17 | 16 | 13 | 15 | 19 | 9 | 7 | 5 | 7 |
| | | | | GH93 exo-α-L-1,5-arabinanase | MYCTH_104827 | AraGH93A | *Myceliophthora thermophila ATCC 42464* | 347008004 | 42 | 9 | 5 | 2 | 2 | 3 | 23 | 10 | 22 | 14 | 12 | 10 | 5 | 8 | 6 | 5 | 7 | 4 | 6 | 5 | 4 | 8 | 4 | 5 | 3 | 4 |
| | - | ? | - | GH95 fucosidase A | MYCTH_101972 | FucGH95A | *Myceliophthora thermophila ATCC 42464* | 347010011 | 91 | 0 | 0 | 0 | 0 | 0 | 23 | 10 | 17 | 10 | 10 | 16 | 8 | 0 | 0 | 4 | 0 | 0 | 0 | 0 | 0 | 3 | 1 | 4 | 3 | 2 |
| | - | GMC_oxred_c | - | GMC oxidoreductase A | MYCTH_2299749 | GloAA3A | *Myceliophthora thermophila ATCC 42464* | 347008190 | 69 | 17 | 9 | 7 | 7 | 8 | 19 | 8 | 20 | 12 | 10 | 34 | 17 | 18 | 13 | 15 | 11 | 6 | 15 | 12 | 9 | 20 | 9 | 15 | 10 | 10 |
| | - | Pec_lyase_c | - | PL1 polysaccharide lyase | MYCTH_52463 | PeLPL1A | *Myceliophthora thermophila ATCC 42464* | 347011254 | 34 | 14 | 7 | 12 | 12 | 10 | 34 | 14 | 28 | 17 | 16 | 34 | 17 | 25 | 19 | 18 | 24 | 13 | 16 | 13 | 13 | 16 | 7 | 12 | 8 | 8 |
| | - | peptidase S9 | - | protease, aminopeptidase C | AMPC | | *Myceliophthora thermophila ATCC 42464* | 347005727 | 80 | 11 | 6 | 0 | 0 | 3 | 46 | 20 | 25 | 15 | 17 | 26 | 13 | 8 | 6 | 9 | 31 | 17 | 8 | 6 | 12 | 42 | 20 | 0 | 0 | 10 |
| | | | | thioredoxin reductase | MYCTH_112714 | | *Myceliophthora thermophila ATCC 42464* | 347013124 | 45 | 26 | 13 | 0 | 0 | 7 | 55 | 23 | 0 | 0 | 12 | 33 | 16 | 0 | 0 | 8 | 37 | 21 | 4 | 3 | 12 | 47 | 22 | 0 | 0 | 11 |
| | - | - | WSC | unknown protein | MYCTH_2310464 | | *Myceliophthora thermophila ATCC 42464* | 347013340 | 38 | 19 | 10 | 9 | 9 | 10 | 12 | 5 | 0 | 0 | 3 | 16 | 8 | 14 | 10 | 9 | 4 | 2 | 0 | 0 | 1 | 18 | 8 | 9 | 6 | 7 |
| | - | DUF | - | unknown protein | MYCTH_101354 | | *Myceliophthora thermophila ATCC 42464* | 347010721 | 46 | 3 | 2 | 0 | 0 | 1 | 0 | 0 | 0 | 0 | 0 | 12 | 6 | 11 | 8 | 7 | 5 | 3 | 5 | 4 | 3 | 18 | 8 | 12 | 8 | 8 |
| | - | NPP1 | - | unknown protein | MYCTH_108493 | | *Myceliophthora thermophila ATCC 42464* | 347012790 | 33 | 5 | 3 | 0 | 0 | 1 | 17 | 7 | 4 | 2 | 5 | 15 | 7 | 9 | 7 | 7 | 4 | 2 | 2 | 2 | 2 | 14 | 7 | 7 | 5 | 6 |
| | | | | unknown protein | MYCTH_2299856 | | *Myceliophthora thermophila ATCC 42464* | 347008243 | 42 | 12 | 6 | 10 | 10 | 8 | 11 | 5 | 9 | 6 | 5 | 10 | 5 | 8 | 6 | 5 | 16 | 9 | 8 | 6 | 8 | 16 | 7 | 9 | 6 | 7 |
| | | | | unknown protein | MYCTH_2301831 | | *Myceliophthora thermophila ATCC 42464* | 347009183 | 33 | 4 | 2 | 0 | 0 | 1 | 3 | 1 | 5 | 3 | 2 | 7 | 3 | 6 | 4 | 4 | 3 | 2 | 6 | 5 | 3 | 9 | 4 | 13 | 9 | 6 |
| | | | | unknown protein | MYCTH_2306121 | | *Myceliophthora thermophila ATCC 42464* | 347011160 | 32 | 15 | 8 | 5 | 5 | 6 | 12 | 5 | 10 | 6 | 6 | 5 | 2 | 5 | 4 | 3 | 6 | 3 | 5 | 4 | 4 | 2 | 1 | 0 | 0 | 0 |
| | | | | unknown protein | MYCTH_64095 | | *Myceliophthora thermophila ATCC 42464* | 347007100 | 105 | 0 | 0 | 0 | 0 | 0 | 23 | 10 | 7 | 4 | 7 | 0 | 0 | 0 | 0 | 0 | 0 | 0 | 0 | 0 | 0 | 0 | 0 | 0 | 0 | 0 |
| | | | | unknown protein | MYCTH_2299664 | | *Myceliophthora thermophila ATCC 42464* | 347008141 | 49 | 10 | 5 | 4 | 4 | 5 | 0 | 0 | 0 | 0 | 0 | 0 | 0 | 0 | 0 | 0 | 10 | 6 | 18 | 14 | 10 | 21 | 10 | 0 | 0 | 5 |
| | | ? | - | unknown protein A (glutaminase) | MYCTH_2307339 | | *Myceliophthora thermophila ATCC 42464* | 347011745 | 76 | 47 | 24 | 13 | 13 | 19 | 43 | 18 | 47 | 29 | 24 | 36 | 18 | 26 | 19 | 19 | 48 | 27 | 33 | 26 | 27 | 49 | 23 | 32 | 21 | 22 |
| | | | | unknown protein B | MYCTH_2307666 | | *Myceliophthora thermophila ATCC 42464* | 347011921 | 55 | 16 | 8 | 16 | 16 | 12 | 17 | 7 | 21 | 13 | 10 | 19 | 9 | 17 | 13 | 11 | 24 | 13 | 22 | 18 | 15 | 15 | 7 | 17 | 11 | 9 |
| | - | - | CBM1 | unknown protein C | MYCTH_33936 | | *Myceliophthora thermophila ATCC 42464* | 347012789 | 32 | 14 | 7 | 13 | 13 | 10 | 13 | 6 | 7 | 4 | 5 | 13 | 6 | 13 | 10 | 8 | 10 | 6 | 11 | 9 | 7 | 11 | 5 | 13 | 9 | 7 |
| oxred | | FAD binding | - | unknown protein D | MYCTH_2126295 | | *Myceliophthora thermophila ATCC 42464* | 347009809 | 52 | 9 | 5 | 5 | 5 | 5 | 22 | 9 | 18 | 11 | 10 | 19 | 9 | 16 | 12 | 11 | 9 | 5 | 14 | 11 | 8 | 25 | 12 | 24 | 16 | 14 |
| Lower tier of secreted proteins (<25% of N-TSC in each condition) | | | | | | | | | | | | | | | | | | | | | | | | | | | | | | | | | | |
| | Cu_oxi | Cu_oxi | Cu_oxi | AA1 laccase | MYCTH_99853 | LacA | *Myceliophthora thermophila ATCC 42464* | 347007110 | 66 | 4 | 2 | 0 | 0 | 1 | 7 | 3 | 5 | 3 | 3 | 6 | 3 | 7 | 5 | 4 | 2 | 1 | 0 | 0 | 1 | 3 | 1 | 6 | 4 | 3 |
| | Cu_oxi | Cu_oxi | Cu_oxi | AA1 multicopper oxidase | MYCTH_75296 | LacB | *Myceliophthora thermophila ATCC 42464* | 347013702 | 72 | 5 | 3 | 0 | 0 | 1 | 10 | 4 | 7 | 4 | 4 | 11 | 5 | 4 | 3 | 4 | 3 | 2 | 0 | 0 | 1 | 2 | 1 | 7 | 5 | 3 |
| | - | NAD binding | - | AA3 cellobiose dehydrogenase C | MYCTH_58125 | CdhAA3C | *Myceliophthora thermophila ATCC 42464* | 347013821 | 57 | 0 | 0 | 0 | 0 | 0 | 2 | 1 | 0 | 0 | 0 | 5 | 2 | 0 | 0 | 1 | 5 | 3 | 0 | 0 | 1 | 2 | 1 | 0 | 0 | 0 |
| | - | NAD binding | - | AA3 cellobiose dehydrogenase D | MYCTH_102971 | CdhAA3D | *Myceliophthora thermophila ATCC 42464* | 347011028 | 54 | 4 | 2 | 0 | 0 | 1 | 4 | 2 | 0 | 0 | 1 | 7 | 3 | 0 | 0 | 2 | 0 | 0 | 0 | 0 | 0 | 5 | 2 | 0 | 0 | 1 |
| | - | GH_61 | - | AA9 lytic polysaccharide monooxygenase C | MYCTH_79765 | LpmoAA9C | *Myceliophthora thermophila ATCC 42464* | 347008529 | 26 | 6 | 3 | 3 | 3 | 3 | 0 | 0 | 0 | 0 | 0 | 0 | 0 | 0 | 0 | 0 | 0 | 0 | 0 | 0 | 0 | 0 | 0 | 0 | 0 | 0 |
| | - | GH_61 | - | AA9 lytic polysaccharide monooxygenase D | MYCTH_92668 | LpmoAA9D | *Myceliophthora thermophila ATCC 42464* | 347009178 | 27 | 7 | 4 | 0 | 0 | 2 | 0 | 0 | 0 | 0 | 0 | 0 | 0 | 0 | 0 | 0 | 7 | 4 | 0 | 0 | 2 | 3 | 1 | 3 | 2 | 2 |
| | - | GH_61 | CBM1 | AA9 lytic polysaccharide monooxygenase E | MYCTH_111088 | LpmoAA9E | *Myceliophthora thermophila ATCC 42464* | 347008929 | 31 | 0 | 0 | 3 | 3 | 2 | 0 | 0 | 0 | 0 | 0 | 3 | 1 | 0 | 0 | 1 | 0 | 0 | 0 | 0 | 0 | 0 | 0 | 0 | 0 | 0 |
| | - | GH_61 | - | AA9 lytic polysaccharide monooxygenase F | MYCTH_85556 | LpmoAA9F | *Myceliophthora thermophila ATCC 42464* | 347013822 | 24 | 0 | 0 | 2 | 2 | 1 | 0 | 0 | 0 | 0 | 0 | 3 | 1 | 0 | 0 | 1 | 0 | 0 | 0 | 0 | 0 | 0 | 0 | 0 | 0 | 0 |
| | - | GH_61 | - | AA9 lytic polysaccharide monooxygenase G | MYCTH_2112799 | LpmoAA9G | *Myceliophthora thermophila ATCC 42464* | 347013774 | 26 | 3 | 2 | 0 | 0 | 1 | 0 | 0 | 0 | 0 | 0 | 4 | 2 | 0 | 0 | 1 | 0 | 0 | 0 | 0 | 0 | 0 | 0 | 0 | 0 | 0 |
| | | | | actin | | | *Thielavia terrestris NRRL 8126* | 346997015 | 42 | 0 | 0 | 0 | 0 | 0 | 0 | 0 | 0 | 0 | 0 | 0 | 0 | 4 | 3 | 1 | 0 | 0 | 0 | 0 | 0 | 0 | 0 | 0 | 0 | 0 |
| | BBE | FAD binding | - | alcohol oxidase A | MYCTH_2309690 | AloA | *Myceliophthora thermophila ATCC 42464* | 347012985 | 65 | 0 | 0 | 0 | 0 | 0 | 0 | 0 | 0 | 0 | 0 | 0 | 0 | 6 | 4 | 2 | 0 | 0 | 0 | 0 | 0 | 0 | 0 | 0 | 0 | 0 |
| | | | | CE1 carbohydrate esterase | MYCTH_39279 | | *Myceliophthora thermophila ATCC 42464* | 347013611 | 29 | 0 | 0 | 0 | 0 | 0 | 7 | 3 | 0 | 0 | 1 | 11 | 5 | 5 | 4 | 5 | 0 | 0 | 0 | 0 | 0 | 6 | 3 | 0 | 0 | 1 |
| | | | | CE1 carbohydrate esterase | | | *Myceliophthora thermophila ATCC 42464* | 347010761 | 34 | 0 | 0 | 0 | 0 | 0 | 0 | 0 | 0 | 0 | 0 | 4 | 2 | 0 | 0 | 1 | 0 | 0 | 0 | 0 | 0 | 4 | 2 | 0 | 0 | 1 |
| | | | | CE1 carbohydrate esterase | | | *Myceliophthora thermophila ATCC 42464* | 347014526 | 31 | 0 | 0 | 0 | 0 | 0 | 0 | 0 | 0 | 0 | 0 | 3 | 1 | 0 | 0 | 1 | 0 | 0 | 0 | 0 | 0 | 0 | 0 | 0 | 0 | 0 |
| | | | | CE1 ferulic acid esterase A | MYCTH_78090 | FaeCE1A | *Myceliophthora thermophila ATCC 42464* | 347009716 | 31 | 0 | 0 | 0 | 0 | 0 | 0 | 0 | 0 | 0 | 0 | 10 | 5 | 7 | 5 | 5 | 0 | 0 | 0 | 0 | 0 | 4 | 2 | 5 | 3 | 3 |
| | | | | CE16 carbohydrate esterase | | | *Myceliophthora thermophila ATCC 42464* | 347012118 | 32 | 8 | 4 | 7 | 7 | 6 | 0 | 0 | 0 | 0 | 0 | 4 | 2 | 4 | 3 | 2 | 0 | 0 | 4 | 3 | 2 | 5 | 2 | 7 | 5 | 3 |
| | | | | CE3 carbohydrate esterase | MYCTH_105128 | | *Myceliophthora thermophila ATCC 42464* | 347013486 | 32 | 19 | 10 | 0 | 0 | 5 | 11 | 5 | 2 | 1 | 3 | 17 | 8 | 0 | 0 | 4 | 5 | 3 | 0 | 0 | 1 | 3 | 1 | 0 | 0 | 1 |
| | | | | GH1 glycoside hydrolase | | | *Myceliophthora thermophila ATCC 42464* | 347009973 | 54 | 9 | 5 | 0 | 0 | 2 | 0 | 0 | 0 | 0 | 0 | 11 | 5 | 0 | 0 | 3 | 12 | 7 | 0 | 0 | 3 | 17 | 8 | 3 | 2 | 5 |
| | - | GH_10 | - | GH10 endo-1,4-beta-xylanase B | MYCTH_116553 | XynGH10B | *Myceliophthora thermophila ATCC 42464* | 347011113 | 42 | 11 | 6 | 5 | 5 | 5 | 6 | 3 | 5 | 3 | 3 | 9 | 4 | 5 | 4 | 4 | 0 | 0 | 0 | 0 | 0 | 0 | 0 | 0 | 0 | 0 |
| | - | GH_11 | CBM1 | GH11 endo-1,4-beta-xylanase B | MYCTH_100068 | XynGH11B | *Myceliophthora thermophila ATCC 42464* | 367026235 | 29 | 0 | 0 | 0 | 0 | 0 | 0 | 0 | 0 | 0 | 0 | 7 | 3 | 3 | 2 | 3 | 3 | 2 | 3 | 2 | 2 | 3 | 1 | 5 | 3 | 2 |
| | - | GH_11 | - | GH11 endo-1,4-beta-xylanase C | MYCTH_49824 | XynGH11C | *Myceliophthora thermophila ATCC 42464* | 367028490 | 25 | 0 | 0 | 0 | 0 | 0 | 5 | 2 | 0 | 0 | 1 | 4 | 2 | 0 | 0 | 1 | 0 | 0 | 0 | 0 | 0 | 0 | 0 | 0 | 0 | 0 |
| | - | GH_11 | - | GH11 endo-1,4-beta-xylanase G | MYCTH_99786 | XynGH11G | *Myceliophthora thermophila ATCC 42464* | 367020944 | 24 | 4 | 2 | 0 | 0 | 1 | 5 | 2 | 3 | 2 | 2 | 2 | 1 | 4 | 3 | 2 | 2 | 1 | 4 | 3 | 2 | 2 | 1 | 0 | 0 | 0 |
| | | | | GH12 glycoside hydrolase | | | *Myceliophthora thermophila ATCC 42464* | 347013049 | 27 | 0 | 0 | 3 | 3 | 2 | 3 | 1 | 9 | 6 | 3 | 4 | 2 | 3 | 2 | 2 | 0 | 0 | 3 | 2 | 1 | 0 | 0 | 2 | 1 | 1 |
| | | | | GH13 glycoside hydrolase | | | *Myceliophthora thermophila ATCC 42464* | 347006707 | 68 | 0 | 0 | 0 | 0 | 0 | 7 | 3 | 3 | 2 | 2 | 0 | 0 | 0 | 0 | 0 | 5 | 3 | 0 | 0 | 1 | 0 | 0 | 0 | 0 | 0 |
| | | | | GH16 glycoside hydrolase | MYCTH_90182 | GH16A | *Myceliophthora thermophila ATCC 42464* | 347013280 | 31 | 7 | 4 | 6 | 6 | 5 | 14 | 6 | 12 | 7 | 7 | 9 | 4 | 8 | 6 | 5 | 8 | 4 | 7 | 6 | 5 | 11 | 5 | 9 | 6 | 6 |
| | | | | GH2 glycoside hydrolase | | | *Myceliophthora thermophila ATCC 42464* | 347011950 | 96 | 0 | 0 | 0 | 0 | 0 | 0 | 0 | 0 | 0 | 0 | 3 | 1 | 0 | 0 | 1 | 0 | 0 | 0 | 0 | 0 | 0 | 0 | 0 | 0 | 0 |
| | | | | GH3 glycoside hydrolase | MYCTH_104628 | GH3B | *Myceliophthora thermophila ATCC 42464* | 347013048 | 90 | 3 | 2 | 0 | 0 | 1 | 13 | 6 | 7 | 4 | 5 | 7 | 3 | 6 | 4 | 4 | 3 | 2 | 5 | 4 | 3 | 8 | 4 | 7 | 5 | 4 |
| | | | | GH31 glycoside hydrolase | | | *Thielavia terrestris NRRL 8126* | 347000863 | 95 | 0 | 0 | 0 | 0 | 0 | 17 | 7 | 0 | 0 | 4 | 0 | 0 | 0 | 0 | 0 | 0 | 0 | 0 | 0 | 0 | 0 | 0 | 0 | 0 | 0 |
| | | | | GH43 glycoside hydrolase | MYCTH_2306666 | GH43B | *Myceliophthora thermophila ATCC 42464* | 347011434 | 39 | 5 | 3 | 0 | 0 | 1 | 19 | 8 | 3 | 2 | 5 | 10 | 5 | 8 | 6 | 5 | 0 | 0 | 0 | 0 | 0 | 0 | 0 | 0 | 0 | 0 |
| | | | | GH43 glycoside hydrolase | MYCTH_39555 | | *Myceliophthora thermophila ATCC 42464* | 347013594 | 37 | 2 | 1 | 0 | 0 | 1 | 4 | 2 | 0 | 0 | 1 | 10 | 5 | 0 | 0 | 2 | 0 | 0 | 0 | 0 | 0 | 14 | 7 | 0 | 0 | 3 |
| | | | | GH45 glycoside hydrolase | | | *Myceliophthora thermophila ATCC 42464* | 347009915 | 47 | 0 | 0 | 0 | 0 | 0 | 0 | 0 | 0 | 0 | 0 | 0 | 0 | 3 | 2 | 1 | 0 | 0 | 0 | 0 | 0 | 0 | 0 | 0 | 0 | 0 |
| | | | | GH45 glycoside hydrolase | | | *Myceliophthora thermophila ATCC 42464* | 347006590 | 24 | 0 | 0 | 4 | 4 | 2 | 0 | 0 | 0 | 0 | 0 | 0 | 0 | 0 | 0 | 0 | 6 | 3 | 6 | 5 | 4 | 6 | 3 | 7 | 5 | 4 |
| | | | | GH5 glycoside hydrolase | | | *Myceliophthora thermophila ATCC 42464* | 347010970 | 37 | 4 | 2 | 5 | 5 | 4 | 7 | 3 | 4 | 2 | 3 | 0 | 0 | 0 | 0 | 0 | 6 | 3 | 0 | 0 | 2 | 0 | 0 | 0 | 0 | 0 |
| | | | | GH51 glycoside hydrolase | | | *Myceliophthora thermophila ATCC 42464* | 347010967 | 58 | 0 | 0 | 0 | 0 | 0 | 0 | 0 | 0 | 0 | 0 | 3 | 1 | 0 | 0 | 1 | 0 | 0 | 0 | 0 | 0 | 0 | 0 | 0 | 0 | 0 |
| | | | | GH55 glycoside hydrolase | | | *Myceliophthora thermophila ATCC 42464* | 347012862 | 82 | 7 | 4 | 3 | 3 | 3 | 0 | 0 | 0 | 0 | 0 | 0 | 0 | 0 | 0 | 0 | 8 | 4 | 4 | 3 | 4 | 5 | 2 | 0 | 0 | 1 |
| | | | | GH72 glycoside hydrolase | MYCTH_66349 | GH72 | *Myceliophthora thermophila ATCC 42464* | 347008543 | 49 | 12 | 6 | 0 | 0 | 3 | 11 | 5 | 0 | 0 | 2 | 16 | 8 | 0 | 0 | 4 | 23 | 13 | 0 | 0 | 6 | 9 | 4 | 0 | 0 | 2 |
| | | | | GH72 glycoside hydrolase | | | *Myceliophthora thermophila ATCC 42464* | 347014483 | 52 | 7 | 4 | 0 | 0 | 2 | 0 | 0 | 0 | 0 | 0 | 5 | 2 | 0 | 0 | 1 | 3 | 2 | 0 | 0 | 1 | 14 | 7 | 0 | 0 | 3 |
| | | | | GH72 glycoside hydrolase | | | *Myceliophthora thermophila ATCC 42464* | 347013570 | 50 | 0 | 0 | 0 | 0 | 0 | 0 | 0 | 0 | 0 | 0 | 5 | 2 | 0 | 0 | 1 | 0 | 0 | 0 | 0 | 0 | 7 | 3 | 0 | 0 | 2 |
| | | | | GH75 glycoside hydrolase | | | *Myceliophthora thermophila ATCC 42464* | 347013426 | 33 | 0 | 0 | 0 | 0 | 0 | 0 | 0 | 4 | 2 | 1 | 5 | 2 | 4 | 3 | 3 | 0 | 0 | 0 | 0 | 0 | 6 | 3 | 7 | 5 | 4 |
| | | | | GH79 glycoside hydrolase | | | *Myceliophthora thermophila ATCC 42464* | 347005644 | 40 | 5 | 3 | 0 | 0 | 1 | 2 | 1 | 0 | 0 | 0 | 0 | 0 | 0 | 0 | 0 | 0 | 0 | 0 | 0 | 0 | 0 | 0 | 0 | 0 | 0 |
| | | | | GH93 glycoside hydrolase | | | *Myceliophthora thermophila ATCC 42464* | 347012961 | 46 | 0 | 0 | 0 | 0 | 0 | 4 | 2 | 0 | 0 | 1 | 8 | 4 | 4 | 3 | 3 | 0 | 0 | 0 | 0 | 0 | 0 | 0 | 0 | 0 | 0 |
| | PRK11 | GMC_oxred_c | - | glucose oxidase | MYCTH_2305709 | GloAA3B | *Myceliophthora thermophila ATCC 42464* | 347010915 | 69 | 0 | 0 | 0 | 0 | 0 | 0 | 0 | 0 | 0 | 0 | 6 | 3 | 0 | 0 | 1 | 0 | 0 | 0 | 0 | 0 | 5 | 2 | 0 | 0 | 1 |
| | - | GMC_oxred_c | - | GMC oxidoreductase | | | *Myceliophthora thermophila ATCC 42464* | 347011121 | 68 | 0 | 0 | 0 | 0 | 0 | 0 | 0 | 0 | 0 | 0 | 9 | 4 | 0 | 0 | 2 | 0 | 0 | 0 | 0 | 0 | 10 | 5 | 0 | 0 | 2 |
| | Rossmann | GMC_oxred_c | - | GMC oxidoreductase | | | *Myceliophthora thermophila ATCC 42464* | 347013781 | 71 | 0 | 0 | 0 | 0 | 0 | 0 | 0 | 0 | 0 | 0 | 6 | 3 | 0 | 0 | 1 | 0 | 0 | 0 | 0 | 0 | 0 | 0 | 0 | 0 | 0 |
| | DUF2280 | - | - | Intradiol ring-cleavage dioxygenase | | | *Myceliophthora thermophila ATCC 42464* | 347013137 | 41 | 0 | 0 | 0 | 0 | 0 | 0 | 0 | 2 | 1 | 1 | 0 | 0 | 0 | 0 | 0 | 0 | 0 | 0 | 0 | 0 | 0 | 0 | 0 | 0 | 0 |
| | - | GFO_IDH_MocA | - | NADP:D-xylose dehydrogenase | | | *Myceliophthora thermophila ATCC 42464* | 347014406 | 43 | 0 | 0 | 0 | 0 | 0 | 0 | 0 | 0 | 0 | 0 | 0 | 0 | 0 | 0 | 0 | 0 | 0 | 0 | 0 | 0 | 8 | 4 | 0 | 0 | 2 |
| | | | | phospholipase C | | | *Myceliophthora thermophila ATCC 42464* | 347009837 | 67 | 0 | 0 | 0 | 0 | 0 | 0 | 0 | 0 | 0 | 0 | 7 | 3 | 5 | 4 | 4 | 0 | 0 | 0 | 0 | 0 | 0 | 0 | 0 | 0 | 0 |
| | | | | PL1 polysaccharide lyase | MYCTH_97932 | PelPL1B | *Myceliophthora thermophila ATCC 42464* | 347007508 | 35 | 6 | 3 | 0 | 0 | 2 | 5 | 2 | 6 | 4 | 3 | 7 | 3 | 4 | 3 | 3 | 0 | 0 | 0 | 0 | 0 | 3 | 1 | 0 | 0 | 1 |
| | | | | PL20 polysaccharide lyase | | | *Myceliophthora thermophila ATCC 42464* | 347011988 | 29 | 0 | 0 | 0 | 0 | 0 | 0 | 0 | 0 | 0 | 0 | 2 | 1 | 4 | 3 | 2 | 0 | 0 | 0 | 0 | 0 | 2 | 1 | 0 | 0 | 0 |
| | | | | PL3 polysaccharide lyase | | | *Myceliophthora thermophila ATCC 42464* | 347011849 | 26 | 0 | 0 | 0 | 0 | 0 | 3 | 1 | 3 | 2 | 2 | 0 | 0 | 0 | 0 | 0 | 0 | 0 | 0 | 0 | 0 | 0 | 0 | 0 | 0 | 0 |
| | | | | PL4 polysaccharide lyase | | | *Myceliophthora thermophila ATCC 42464* | 347011711 | 58 | 0 | 0 | 0 | 0 | 0 | 0 | 0 | 0 | 0 | 0 | 0 | 0 | 4 | 3 | 1 | 0 | 0 | 10 | 8 | 4 | 0 | 0 | 0 | 0 | 0 |
| | - | tyrosinase | - | polyphenol oxidase | | | *Myceliophthora thermophila ATCC 42464* | 347013282 | 46 | 4 | 2 | 4 | 4 | 3 | 0 | 0 | 4 | 2 | 1 | 0 | 0 | 4 | 3 | 1 | 0 | 0 | 7 | 6 | 3 | 0 | 0 | 3 | 2 | 1 |
| | - | tyrosinase | - | polyphenol oxidase | | | *Myceliophthora thermophila ATCC 42464* | 347011242 | 37 | 12 | 6 | 0 | 0 | 3 | 0 | 0 | 0 | 0 | 0 | 0 | 0 | 0 | 0 | 0 | 0 | 0 | 0 | 0 | 0 | 0 | 0 | 0 | 0 | 0 |
| | In_I9 | peptidase S8_53 | - | protease, carboxypeptidase | | | *Myceliophthora thermophila ATCC 42464* | 347009682 | 40 | 0 | 0 | 0 | 0 | 0 | 0 | 0 | 0 | 0 | 0 | 0 | 0 | 0 | 0 | 0 | 0 | 0 | 3 | 2 | 1 | 0 | 0 | 0 | 0 | 0 |
| | | | | protease, carboxypeptidase | | | *Myceliophthora thermophila ATCC 42464* | 347009113 | 46 | 3 | 2 | 0 | 0 | 1 | 10 | 4 | 8 | 5 | 5 | 0 | 0 | 0 | 0 | 0 | 0 | 0 | 0 | 0 | 0 | 0 | 0 | 0 | 0 | 0 |
| | Zi | PA | Zn-pep | protease, aminopeptidase A | | | *Myceliophthora thermophila ATCC 42464* | 347014627 | 54 | 12 | 6 | 4 | 4 | 5 | 0 | 0 | 0 | 0 | 0 | 6 | 3 | 0 | 0 | 1 | 0 | 0 | 0 | 0 | 0 | 0 | 0 | 0 | 0 | 0 |
| | Zi | PA | Zn-pep | protease, aminopeptidase B | | | *Myceliophthora thermophila ATCC 42464* | 347012974 | 53 | 12 | 6 | 0 | 0 | 3 | 0 | 0 | 0 | 0 | 0 | 3 | 1 | 0 | 0 | 1 | 0 | 0 | 0 | 0 | 0 | 0 | 0 | 0 | 0 | 0 |
| | propep | peptidase S8_53 | - | protease, aminopeptidase D | | | *Myceliophthora thermophila ATCC 42464* | 347009020 | 67 | 3 | 2 | 0 | 0 | 1 | 0 | 0 | 0 | 0 | 0 | 0 | 0 | 0 | 0 | 0 | 0 | 0 | 0 | 0 | 0 | 0 | 0 | 0 | 0 | 0 |
| | - | - | CBM | unknown protein | MYCTH_112399 | | *Myceliophthora thermophila ATCC 42464* | 347014594 | 37 | 0 | 0 | 0 | 0 | 0 | 0 | 0 | 0 | 0 | 0 | 7 | 3 | 5 | 4 | 4 | 0 | 0 | 0 | 0 | 0 | 4 | 2 | 6 | 4 | 3 |
| | | | | unknown protein | MYCTH_2297816 | | *Myceliophthora thermophila ATCC 42464* | 347007306 | 16 | 6 | 3 | 0 | 0 | 2 | 6 | 3 | 0 | 0 | 1 | 9 | 4 | 2 | 1 | 3 | 0 | 0 | 0 | 0 | 0 | 0 | 0 | 0 | 0 | 0 |
| | | | | unknown protein | MYCTH_2295992 | | *Myceliophthora thermophila ATCC 42464* | 347006501 | 98 | 11 | 6 | 0 | 0 | 3 | 6 | 3 | 0 | 0 | 1 | 11 | 5 | 0 | 0 | 3 | 10 | 6 | 3 | 2 | 4 | 11 | 5 | 0 | 0 | 3 |
| | | | | unknown protein | MYCTH_102138 | | *Myceliophthora thermophila ATCC 42464* | 347009818 | 20 | 4 | 2 | 3 | 3 | 3 | 4 | 2 | 0 | 0 | 1 | 3 | 1 | 4 | 3 | 2 | 0 | 0 | 0 | 0 | 0 | 0 | 0 | 0 | 0 | 0 |
| | | | | unknown protein | MYCTH_2300931 | | *Myceliophthora thermophila ATCC 42464* | 347008746 | 110 | 0 | 0 | 0 | 0 | 0 | 0 | 0 | 0 | 0 | 0 | 4 | 2 | 2 | 1 | 2 | 0 | 0 | 0 | 0 | 0 | 5 | 2 | 0 | 0 | 1 |
| | | | | unknown protein | MYCTH_2309042 | | *Myceliophthora thermophila ATCC 42464* | 347012661 | 75 | 0 | 0 | 0 | 0 | 0 | 9 | 4 | 0 | 0 | 2 | 6 | 3 | 0 | 0 | 1 | 3 | 2 | 0 | 0 | 1 | 0 | 0 | 0 | 0 | 0 |
| | | | | unknown protein | MYCTH_2299483 | | *Myceliophthora thermophila ATCC 42464* | 347008045 | 69 | 0 | 0 | 0 | 0 | 0 | 8 | 3 | 3 | 2 | 3 | 5 | 2 | 0 | 0 | 1 | 8 | 4 | 2 | 2 | 3 | 3 | 1 | 4 | 3 | 2 |
| | | | | unknown protein | MYCTH_2303093 | | *Myceliophthora thermophila ATCC 42464* | 347009730 | 62 | 4 | 2 | 0 | 0 | 1 | 6 | 3 | 3 | 2 | 2 | 5 | 2 | 0 | 0 | 1 | 4 | 2 | 0 | 0 | 1 | 5 | 2 | 0 | 0 | 1 |
| | | | | unknown protein | MYCTH_75329 | | *Myceliophthora thermophila ATCC 42464* | 347013738 | 20 | 0 | 0 | 0 | 0 | 0 | 5 | 2 | 0 | 0 | 1 | 5 | 2 | 0 | 0 | 1 | 4 | 2 | 0 | 0 | 1 | 0 | 0 | 0 | 0 | 0 |
| | | | | unknown protein | MYCTH_2307988 | | *Myceliophthora thermophila ATCC 42464* | 347012092 | 27 | 0 | 0 | 0 | 0 | 0 | 0 | 0 | 0 | 0 | 0 | 0 | 0 | 2 | 1 | 1 | 0 | 0 | 4 | 3 | 2 | 0 | 0 | 4 | 3 | 1 |
| | | | | unknown protein | MYCTH_2122605 | | *Myceliophthora thermophila ATCC 42464* | 347006248 | 42 | 0 | 0 | 0 | 0 | 0 | 0 | 0 | 0 | 0 | 0 | 3 | 1 | 0 | 0 | 1 | 0 | 0 | 0 | 0 | 0 | 3 | 1 | 0 | 0 | 1 |
| | - | DUF1237 | - | unknown protein | MYCTH_59997 | | *Myceliophthora thermophila ATCC 42464* | 347007677 | 61 | 0 | 0 | 0 | 0 | 0 | 6 | 3 | 0 | 0 | 1 | 3 | 1 | 0 | 0 | 1 | 0 | 0 | 0 | 0 | 0 | 0 | 0 | 0 | 0 | 0 |
| | | | | unknown protein | MYCTH_2303940 | | *Myceliophthora thermophila ATCC 42464* | 347010116 | 51 | 0 | 0 | 0 | 0 | 0 | 5 | 2 | 0 | 0 | 1 | 3 | 1 | 0 | 0 | 1 | 0 | 0 | 0 | 0 | 0 | 0 | 0 | 0 | 0 | 0 |
| | | | | unknown protein | MYCTH_2084531 | | *Myceliophthora thermophila ATCC 42464* | 347013999 | 55 | 0 | 0 | 2 | 2 | 1 | 0 | 0 | 0 | 0 | 0 | 0 | 0 | 0 | 0 | 0 | 2 | 1 | 10 | 8 | 5 | 3 | 1 | 11 | 7 | 4 |
| | | | | unknown protein | MYCTH_2308135 | | *Myceliophthora thermophila ATCC 42464* | 347012190 | 104 | 0 | 0 | 0 | 0 | 0 | 0 | 0 | 7 | 4 | 2 | 0 | 0 | 0 | 0 | 0 | 0 | 0 | 3 | 2 | 1 | 0 | 0 | 8 | 5 | 3 |
| | | | | unknown protein | MYCTH_2301054 | | *Myceliophthora thermophila ATCC 42464* | 347008809 | 32 | 0 | 0 | 0 | 0 | 0 | 0 | 0 | 0 | 0 | 0 | 0 | 0 | 0 | 0 | 0 | 0 | 0 | 4 | 3 | 2 | 0 | 0 | 3 | 2 | 1 |
| | | | | unknown protein | MYCTH_2295704 | | *Myceliophthora thermophila ATCC 42464* | 347006346 | 32 | 4 | 2 | 0 | 0 | 1 | 0 | 0 | 0 | 0 | 0 | 0 | 0 | 0 | 0 | 0 | 4 | 2 | 4 | 3 | 3 | 4 | 2 | 0 | 0 | 1 |
| | | | | unknown protein | MYCTH_2306107 | | *Myceliophthora thermophila ATCC 42464* | 347011151 | 52 | 3 | 2 | 0 | 0 | 1 | 3 | 1 | 0 | 0 | 1 | 0 | 0 | 0 | 0 | 0 | 2 | 1 | 0 | 0 | 1 | 4 | 2 | 0 | 0 | 1 |
| | | | | unknown protein | MYCTH_2302336 | | *Myceliophthora thermophila ATCC 42464* | 347009400 | 65 | 0 | 0 | 0 | 0 | 0 | 0 | 0 | 0 | 0 | 0 | 0 | 0 | 0 | 0 | 0 | 0 | 0 | 0 | 0 | 0 | 4 | 2 | 0 | 0 | 1 |
| | | | | unknown protein | MYCTH_2311687 | | *Myceliophthora thermophila ATCC 42464* | 347014008 | 26 | 2 | 1 | 0 | 0 | 1 | 3 | 1 | 0 | 0 | 1 | 0 | 0 | 0 | 0 | 0 | 0 | 0 | 0 | 0 | 0 | 3 | 1 | 0 | 0 | 1 |
| | | | | unknown protein | MYCTH_2301051 | | *Myceliophthora thermophila ATCC 42464* | 347008807 | 17 | 5 | 3 | 0 | 0 | 1 | 0 | 0 | 0 | 0 | 0 | 0 | 0 | 0 | 0 | 0 | 0 | 0 | 0 | 0 | 0 | 3 | 1 | 0 | 0 | 1 |
| | | | | unknown protein | MYCTH_2296038 | | *Myceliophthora thermophila ATCC 42464* | 347006517 | 30 | 0 | 0 | 0 | 0 | 0 | 4 | 2 | 0 | 0 | 1 | 0 | 0 | 0 | 0 | 0 | 0 | 0 | 0 | 0 | 0 | 2 | 1 | 0 | 0 | 0 |
| | | | | unknown protein | MYCTH_2306748 | | *Myceliophthora thermophila ATCC 42464* | 347011470 | 54 | 5 | 3 | 0 | 0 | 1 | 0 | 0 | 0 | 0 | 0 | 0 | 0 | 0 | 0 | 0 | 4 | 2 | 3 | 2 | 2 | 0 | 0 | 0 | 0 | 0 |
| | | | | unknown protein | MYCTH_2119688 | | *Myceliophthora thermophila ATCC 42464* | 347011992 | 111 | 0 | 0 | 0 | 0 | 0 | 0 | 0 | 0 | 0 | 0 | 0 | 0 | 0 | 0 | 0 | 0 | 0 | 4 | 3 | 2 | 0 | 0 | 0 | 0 | 0 |
| | | | | unknown protein | MYCTH_2311910 | | *Myceliophthora thermophila ATCC 42464* | 347014116 | 50 | 0 | 0 | 0 | 0 | 0 | 0 | 0 | 0 | 0 | 0 | 0 | 0 | 0 | 0 | 0 | 0 | 0 | 2 | 2 | 1 | 0 | 0 | 0 | 0 | 0 |
| | | | | unknown protein | MYCTH_2304102 | | *Myceliophthora thermophila ATCC 42464* | 347010181 | 38 | 0 | 0 | 0 | 0 | 0 | 0 | 0 | 6 | 4 | 2 | 0 | 0 | 0 | 0 | 0 | 0 | 0 | 0 | 0 | 0 | 0 | 0 | 0 | 0 | 0 |
| | | | | unknown protein | MYCTH_80274 | | *Myceliophthora thermophila ATCC 42464* | 347010715 | 89 | 10 | 5 | 0 | 0 | 3 | 7 | 3 | 0 | 0 | 1 | 0 | 0 | 0 | 0 | 0 | 0 | 0 | 0 | 0 | 0 | 0 | 0 | 0 | 0 | 0 |
| | | | | unknown protein | MYCTH_2296814 | | *Myceliophthora thermophila ATCC 42464* | 347006852 | 60 | 0 | 0 | 0 | 0 | 0 | 4 | 2 | 0 | 0 | 1 | 0 | 0 | 0 | 0 | 0 | 0 | 0 | 0 | 0 | 0 | 0 | 0 | 0 | 0 | 0 |
| | | | | unknown protein | MYCTH_2302321 | | *Myceliophthora thermophila ATCC 42464* | 347009397 | 63 | 0 | 0 | 0 | 0 | 0 | 4 | 2 | 0 | 0 | 1 | 0 | 0 | 0 | 0 | 0 | 0 | 0 | 0 | 0 | 0 | 0 | 0 | 0 | 0 | 0 |
| | | | | unknown protein | MYCTH_2297119 | | *Myceliophthora thermophila ATCC 42464* | 347007001 | 63 | 4 | 2 | 5 | 5 | 4 | 0 | 0 | 0 | 0 | 0 | 0 | 0 | 0 | 0 | 0 | 0 | 0 | 0 | 0 | 0 | 0 | 0 | 0 | 0 | 0 |
| | | | | unknown protein | THITE_2110382 | | *Thielavia terrestris NRRL 8126* | 346997931 | 37 | 4 | 2 | 0 | 0 | 1 | 0 | 0 | 0 | 0 | 0 | 0 | 0 | 0 | 0 | 0 | 0 | 0 | 0 | 0 | 0 | 0 | 0 | 0 | 0 | 0 |
| | | | | unknown protein | MYCTH_2297460 | | *Myceliophthora thermophila ATCC 42464* | 347007172 | 38 | 3 | 2 | 0 | 0 | 1 | 0 | 0 | 0 | 0 | 0 | 0 | 0 | 0 | 0 | 0 | 0 | 0 | 0 | 0 | 0 | 0 | 0 | 0 | 0 | 0 |
| | | | | unknown protein | MYCTH_2297118 | | *Myceliophthora thermophila ATCC 42464* | 347007000 | 23 | 2 | 1 | 0 | 0 | 1 | 0 | 0 | 0 | 0 | 0 | 0 | 0 | 0 | 0 | 0 | 0 | 0 | 0 | 0 | 0 | 0 | 0 | 0 | 0 | 0 |
| | | | | unknown protein | MYCTH_2140820 | | *Myceliophthora thermophila ATCC 42464* | 347013943 | 54 | 0 | 0 | 0 | 0 | 0 | 0 | 0 | 0 | 0 | 0 | 0 | 0 | 0 | 0 | 0 | 0 | 0 | 0 | 0 | 0 | 0 | 0 | 0 | 0 | 0 |
| | | | | unknown protein | MYCTH_2303108 | | *Myceliophthora thermophila ATCC 42464* | 347009733 | 42 | 0 | 0 | 0 | 0 | 0 | 0 | 0 | 0 | 0 | 0 | 0 | 0 | 0 | 0 | 0 | 0 | 0 | 0 | 0 | 0 | 0 | 0 | 0 | 0 | 0 |
